# Frequency of Food Intake and Estimated Nutrient Intake among Men and Women: The JACC Study.

**DOI:** 10.2188/jea.15.S24

**Published:** 2005-05-16

**Authors:** Hiroyasu Iso, Chigusa Date, Hiroyuki Noda, Takesumi Yoshimura, Akiko Tamakoshi

**Affiliations:** 1 Department of Public Health Medicine, Doctoral Program in Social and Environmental Medicine, Graduate School of Comprehensive Human Sciences, University of Tsukuba.; 2 Department of Nutrition and Food Science, Faculty of Human Environmental Science, Mukogawa Women’s University.; 3 Fukuoka Institute of Health and Environmental Sciences.; 4 Department of Preventive Medicine/Biostatistics and Medical Decision Making, Nagoya University Graduate School of Medicine.

**Keywords:** food intake, nutrition, Japanese

## Abstract

BACKGROUND: The aim of this study was to determine the frequency of food intake and estimated nutrient intake in the JACC study cohort.

METHODS: The subjects were 46,465 men and 64,327 women aged 40-79 years who responded to the self-administered food frequency questionnaire. We calculated the dietary intake of major nutrients by multiplying the frequency of consumption of each food with each portion size, estimated from a validation study.

RESULTS: Women reported to more likely consume vegetables, seaweed, fruits, sweets, oolong-tea, western-style-breakfast, and less likely to consume rice and miso-soup than men. Women reported less preference of salty foods and fatty foods than men. Compared with men, women had higher mean intakes of carotene and vitamin C, and lower intake of total energy, carbohydrate and sodium. The frequency of consumption of beef, chicken, dairy products, fresh fish, fish products, rice, and miso-soup increased with age in men, and that of vegetables, seaweed, beans, tofu, fruits, sweets, and green-tea increased with age in both sexes. Men aged 40-49years had the lowest mean intake levels of crude fiber, calcium, iron, retinol, carotene, and vitamins A, C, and E. Women aged 40-49years had the lowest mean intake levels of crude fiber, iron, and vitamins C. Women aged 70-79years had the lowest mean intake levels of calcium, retinol, and vitamins A.

CONCLUSIONS: Women had a more westernized dietary pattern than men. Elderly men had a mixture of unhealthy and healthy dietary patterns while elderly women generally had a healthier dietary pattern compared with younger persons.

We provided a baseline food frequency questionnaire of 39 foods to the participants in the Japan Collaborative Cohort Study (JACC Study) for Evaluation of Cancer Risk sponsored by the Ministry of Education, Science, Sports and Culture of Japan (Monbusho). Approximately, 110,000 participants provided valid responses, which enabled us to examine the relationships between food and nutrient intake with risk of mortality from various diseases.

Elucidation of the frequency of food intake and estimation of nutrient intake at baseline would be of value for the hypothesis development and interpretation of the diet-mortality associations. In the present study, we examined the frequency of food and nutrient intake among 110,792 Japanese men and women.

## METHODS

The JACC Study began in 1988-1990 when 110,792 individuals (46,465 men and 64,327 women) aged 40-79 years living in 45 communities across Japan participated in municipal health screening examinations and completed self-administered questionnaires about their lifestyles and medical histories of cardiovascular disease and cancer.

Each participant was asked about the frequency of intake of 35 foods, and five responses were possible for each food item, ranging from “rarely”, “1-2 days/month”, “1-2 days/week”, “3-4 days/week”, and “almost every day”. We calculated the consumption of each food by multiplying the frequency score of consumption of each food 0, 1.5, 3.5, and 7, respectively) with each portion size, estimated from the validation study conducted in 8 men and 77 women from the baseline participants. The average daily intake of nutrients was calculated by multiplying the frequency of consumption of each item by its nutrient content per serving and totaling the nutrient intake for all food items. We obtained the nutrient data for 24,386 men and 37,493 women aged 40-79 years. For each food, the valid number of variables varied due to missing data. The reproducibility and validity of this dietary questionnaire was reported elsewhere.^1^ Statistical analyses were not conducted because of the large sample size in each sex and age category.

We examined the frequency of food intake and the mean intakes of major nutrients according to sex and age groups, geographical areas (Hokkaido, Tohoku, Kanto, Chubu, Kinki, Chugoku and Kyushu), and community features (seaside, plains, and mountains/basins). Seaside communities were 9 sites that faced the sea. Plain communities were 20 sites that did not face the sea and did have any mountains or basins. Mountains/basins communities were 16 sites that were not regarded seaside or plain communities.

Our entire study design was approved in 2000 by the Ethical Board at Nagoya University School of Medicine, where the central secretariat of the JACC study is located.

## RESULTS

Table 1 shows the frequency of food intake according to sex and age groups.

**Table 1.** Frequency of food intake according to sex and age groups.

	Men						Women						
	
	40-49 yrs		50-59yrs		60-69 yrs		70-79yrs		40-49 yrs	50-59yrs		60-69 yrs	70-79yrs
Beef													
No of subjects	8,514		9,867		9,635		4,330		11,358	14,297		13,695	5,837
Rare, %	24.5		24.5		23.2		24.3		27.7	27.6		27.7	32.4
1-2/m, %	38.7		37.5		37.0		33.1		30.6	32.5		32.5	30.0
1-2/w, %	29.1		28.5		30.4		31.9		31.0	29.6		30.0	27.9
3-4/w, %	6.6		8.0		7.9		8.9		9.4	9.1		8.7	8.4
5+/w, %	1.1		1.4		1.5		1.8		1.2	1.3		1.1	1.3

Pork (excluding ham and sausages)													
No of subjects	9,008		10,504		10,414		4,588		12,133	15,535		14,492	6,070
Rare, %	5.9		10.0		10.9		13.9		7.4	11.8		14.9	21.4
1-2/m, %	21.5		24.4		25.5		24.0		14.7	20.8		23.7	23.1
1-2/w, %	49.3		44.4		43.6		43.3		49.4	46.4		43.3	39.2
3-4/w, %	19.6		17.2		16.5		15.3		23.7	17.5		15.2	14.1
5+/w, %	3.7		4.0		3.5		3.5		4.8	3.4		2.9	2.3

Ham													
No of subjects	10,519		11,691		10,991		4,805		13,845	16,846		15,163	6,522
Rare, %	14.2		23.8		25.0		30.3		15.4	24.4		29.9	37.2
1-2/m, %	26.8		25.3		27.1		24.1		23.1	25.0		24.7	22.7
1-2/w, %	40.0		33.5		32.1		30.0		40.7	34.0		30.7	26.9
3-4/w, %	15.4		13.8		12.5		12.5		17.0	13.5		11.8	10.7
5+/w, %	3.6		3.6		3.3		3.2		3.8	3.1		2.8	2.5

Chiken													
No of subjects	9,707		11,162		11,081		5,119		12,875	16,308		15,661	6,914
Rare, %	10.5		9.9		9.4		9.5		7.4	8.6		9.5	11.0
1-2/m, %	28.7		27.9		28.2		26.9		19.4	22.7		24.8	24.2
1-2/w, %	45.1		43.5		43.3		43.2		50.7	46.9		44.9	43.5
3-4/w, %	13.8		16.0		16.5		17.0		19.9	19.2		18.2	18.4
5+/w, %	1.8		2.6		2.6		3.4		2.7	2.7		2.7	3.0

Liver													
No of subjects	9,463		10,462		9,499		4,093		12,581	15,067		13,525	5,696
Rare, %	42.8		43.3		43.8		45.2		49.1	50.2		50.7	54.9
1-2/m, %	39.6		36.3		36.7		33.5		34.2	31.9		32.4	27.4
1-2/w, %	14.2		15.5		14.8		15.2		12.9	13.4		12.3	12.8
3-4/w, %	3.0		4.2		3.9		4.9		3.4	3.7		3.7	3.9
5+/w, %	0.4		0.7		0.9		1.3		0.4	0.7		0.8	1.0

Eggs													
No of subjects	11,251		13,198		12,994		6,167		14,747	18,824		18,228	8,292
Rare, %	1.9		2.0		2.1		2.7		1.8	2.5		2.8	3.6
1-2/m, %	5.2		4.0		4.8		5.5		3.5	3.9		5.3	5.5
1-2/w, %	24.5		20.0		21.0		21.8		20.6	21.4		23.8	24.2
3-4/w, %	30.4		26.8		25.9		24.8		31.6	28.7		27.4	25.8
5+/w, %	38.1		47.2		46.3		45.3		42.5	43.4		40.7	40.9

Milk													
No of subjects	11,145		12,798		12,297		5,669		14,659	18,422		17,437	7,839
Rare, %	21.1		22.3		21.2		20.6		17.3	18.8		18.6	22.2
1-2/m, %	11.3		8.4		7.7		7.4		7.4	6.4		6.1	6.4
1-2/w, %	18.3		14.3		13.8		11.9		17.2	13.4		12.2	11.2
3-4/w, %	16.4		14.4		12.9		10.8		17.9	14.9		13.1	11.2
5+/w, %	33.0		40.6		44.4		49.3		40.2	46.5		49.9	49.1

Yogurt													
No of subjects	9,114		9,925		9,037		3,853		11,987	14,315		13,010	5,542
Rare, %	67.2		70.5		69.2		64.7		46.0	54.3		55.4	58.7
1-2/m, %	17.3		14.4		13.4		13.4		24.6	19.3		17.2	14.7
1-2/w, %	9.3		7.9		8.5		10.2		18.0	14.1		13.5	12.4
3-4/w, %	3.3		3.1		4.1		4.8		7.3	6.5		7.0	6.0
5+/w, %	2.9		4.0		4.9		7.0		4.1	5.9		6.9	8.2

Cheese													
No of subjects	9,425		10,217		9,438		3,971		12,417	14,928		13,271	5,486
Rare, %	45.9		52.4		54.9		57.3		44.4	54.5		59.7	67.1
1-2/m, %	34.3		27.7		24.9		21.2		32.1	25.2		21.1	15.3
1-2/w, %	14.6		13.4		12.7		12.8		16.6	12.9		11.6	10.2
3-4/w, %	3.8		4.3		4.9		5.1		5.0	5.2		4.8	4.1
5+/w, %	1.3		2.2		2.6		3.6		1.9	2.3		2.9	3.3

Butter													
No of subjects	9,388		10,132		9,351		3,904		12,340	14,786		13,093	5,411
Rare, %	46.7		54.1		54.1		55.4		41.8	51.4		56.1	63.4
1-2/m, %	29.5		24.8		22.8		18.9		27.5	23.3		19.7	14.9
1-2/w, %	16.1		13.4		13.3		13.2		18.8	15.2		13.1	11.3
3-4/w, %	4.9		5.1		5.5		6.8		7.6	6.1		5.9	5.1
5+/w, %	3.0		2.6		4.4		5.7		4.3	4.0		5.2	5.4

Margarin													
No of subjects	9,626		10,481		9,450		3,957		12,701	15,249		13,520	5,683
Rare, %	40.7		51.3		50.7		51.0		26.3	39.3		42.6	52.2
1-2/m, %	23.3		18.7		17.0		14.7		20.4	19.0		15.8	12.7
1-2/w, %	20.8		16.5		15.3		15.1		26.2	20.1		18.0	15.2
3-4/w, %	8.4		7.5		7.4		8.5		14.5	10.7		9.8	7.9
5+/w, %	6.9		6.1		9.5		10.8		12.7	10.9		13.8	12.0

Deep-fried foods or *tempura*													
No of subjects	8,620		9,881		9,854		4,374		11,597	14,846		14,253	6,039
Rare, %	2.6		4.1		3.7		4.8		2.3	3.6		4.4	5.9
1-2/m, %	25.7		24.2		24.6		24.3		24.0	25.8		27.6	27.9
1-2/w, %	49.9		46.8		48.7		48.8		50.6	47.6		47.5	47.0
3-4/w, %	17.7		19.8		18.8		17.6		18.6	18.6		16.9	15.7
5+/w, %	4.1		5.1		4.2		4.5		4.5	4.4		3.6	3.5

Fried vegetables													
No of subjects	8,643		10,026		10,059		4,548		11,623	15,082		14,659	6,321
Rare, %	2.9		3.3		3.4		4.4		2.3	2.7		3.7	5.5
1-2/m, %	24.4		17.7		16.3		14.2		18.7	14.7		14.8	16.4
1-2/w, %	47.1		43.1		42.2		39.5		46.9	41.3		40.3	40.0
3-4/w, %	19.4		24.0		24.6		25.2		24.1	27.2		25.8	23.7
5+/w, %	6.2		12.0		13.4		16.7		8.0	14.1		15.5	14.4

Raw fish													
No of subjects	10,324		12,243		12,108		5,654		13,732	17,712		17,067	7,567
Rare, %	1.2		1.6		1.5		2.1		1.2	1.7		2.3	2.9
1-2/m, %	8.3		6.1		7.1		7.8		5.5	5.5		7.0	8.5
1-2/w, %	35.8		29.8		32.0		34.5		33.3	28.5		31.4	33.8
3-4/w, %	34.9		33.5		30.8		31.2		38.2	34.3		32.3	31.5
5+/w, %	19.9		29.1		28.6		24.5		21.8	30.0		27.1	23.3

*Kamaboko* (fish paste)													
No of subjects	8,956		9,824		9,287		4,167		11,669	14,113		13,270	5,772
Rare, %	23.8		25.3		24.5		22.2		18.0	20.2		21.4	22.8
1-2/m, %	38.2		33.1		32.8		31.8		35.2	32.3		32.1	30.0
1-2/w, %	28.0		27.2		28.5		30.1		33.3	30.9		31.1	31.4
3-4/w, %	8.4		10.9		11.1		13.0		11.1	13.2		12.2	12.2
5+/w, %	1.7		3.5		3.1		3.1		2.4	3.4		3.1	3.6

*Himono* (Dried fish or salted fish)													
No of subjects	8,958		10,475		10,367		4,598		12,045	15,560		14,561	6,185
Rare, %	7.1		7.7		9.0		10.9		7.0	8.7		11.6	13.1
1-2/m, %	27.8		22.7		23.5		23.2		25.8	23.4		24.3	22.6
1-2/w, %	41.9		38.5		37.7		36.9		41.7	37.1		35.8	36.7
3-4/w, %	16.9		20.0		19.0		19.1		18.3	20.1		17.9	17.9
5+/w, %	6.4		11.2		10.8		10.0		7.3	10.7		10.4	9.8

Spinach or garland chrysanthemumm													
No of subjects	9,610		11,095		11,161		5,227		12,636	16,142		15,884	7,021
Rare, %	1.7		1.5		1.5		1.4		1.0	0.9		0.8	1.0
1-2/m, %	12.0		9.4		7.8		6.8		7.4	6.5		5.1	5.9
1-2/w, %	35.7		31.0		28.0		26.4		32.1	26.9		25.0	25.3
3-4/w, %	28.9		28.9		28.9		28.6		32.4	30.6		29.4	29.0
5+/w, %	21.7		29.2		33.9		36.8		27.1	35.1		39.6	38.8

Carrot or pumpkin													
No of subjects	8,901		10,435		10,450		4,756		12,061	15,735		15,261	6,644
Rare, %	6.2		5.2		4.2		3.3		1.7	1.5		1.5	1.5
1-2/m, %	23.2		20.0		18.6		16.0		10.9	10.5		10.5	11.2
1-2/w, %	38.3		36.1		36.3		37.1		36.4	33.1		32.3	33.6
3-4/w, %	22.7		24.4		25.1		26.5		33.0	31.9		31.1	30.3
5+/w, %	9.6		14.3		15.8		17.1		17.9	22.9		24.6	23.5

Tomatoes													
No of subjects	9,741		11,141		10,924		4,945		12,858	16,302		15,274	6,708
Rare, %	13.3		13.0		13.0		11.1		10.0	10.2		11.9	11.7
1-2/m, %	31.4		27.0		24.5		21.8		23.3	20.6		19.9	21.0
1-2/w, %	33.3		30.6		29.7		31.0		33.7	29.3		28.0	28.5
3-4/w, %	15.3		18.2		19.8		20.5		20.7	22.0		20.9	21.0
5+/w, %	6.7		11.2		13.0		15.6		12.4	18.0		19.3	17.8

Cabbage or head lettuce													
No of subjects	8,898		10,457		10,520		4,780		12,092	15,706		15,266	6,605
Rare, %	1.1		1.8		2.1		2.8		0.8	1.2		1.8	2.4
1-2/m, %	10.0		10.6		11.0		10.2		5.2	6.6		7.4	8.7
1-2/w, %	36.3		34.3		34.4		33.2		28.6	29.6		30.1	31.2
3-4/w, %	31.9		30.4		29.3		29.2		34.8	31.3		30.7	28.9
5+/w, %	20.7		22.9		23.2		24.6		30.6	31.4		30.1	28.8

Chinese cabbage													
No of subjects	8,509		9,715		9,680		4,322		11,268	14,238		13,673	5,881
Rare, %	4.0		4.7		4.3		4.0		5.6	6.0		4.9	5.6
1-2/m, %	18.8		17.2		15.3		13.7		18.2	16.9		14.5	12.8
1-2/w, %	40.2		36.6		35.3		35.1		37.9	34.0		33.5	33.0
3-4/w, %	25.2		25.2		26.0		27.1		24.7	25.0		25.5	25.7
5+/w, %	11.8		16.3		19.1		20.1		13.6	18.1		21.6	22.9

*Sansai* (Edible wild plants)													
No of subjects	9,625		10,806		10,417		4,495		12,627	15,719		14,420	6,029
Rare, %	40.6		39.0		38.0		39.6		43.9	40.8		40.7	40.9
1-2/m, %	40.2		38.2		37.5		34.1		38.0	36.1		33.9	33.0
1-2/w, %	13.9		14.2		14.0		14.9		11.9	13.6		14.3	15.0
3-4/w, %	4.0		6.1		7.5		7.7		4.6	6.5		7.6	7.0
5+/w, %	1.3		2.6		3.0		3.7		1.6	2.9		3.5	4.1

Fungi (enokidake, shiitake, mushroom)													
No of subjects	8,573		9,870		9,842		4,391		11,525	14,779		14,324	6,061
Rare, %	7.2		6.4		6.8		7.4		4.8	4.3		4.4	6.0
1-2/m, %	36.9		33.3		32.9		30.3		26.0	24.7		24.6	25.0
1-2/w, %	37.8		36.6		35.7		36.0		41.2	38.3		37.9	37.5
3-4/w, %	13.7		16.8		17.5		17.9		20.5	23.1		22.8	21.6
5+/w, %	4.5		6.9		7.1		8.4		7.5	9.6		10.4	9.9

Potatoes													
No of subjects	11,114		12,835		12,504		5,916		14,603	18,542		17,802	8,124
Rare, %	6.8		5.2		3.9		3.3		1.7	1.5		1.5	1.7
1-2/m, %	22.0		18.2		17.4		12.9		11.9	10.9		10.2	10.1
1-2/w, %	38.3		35.7		35.3		33.9		37.8	34.1		33.9	31.8
3-4/w, %	23.0		26.3		26.3		29.8		33.7	33.6		31.8	32.2
5+/w, %	10.0		14.5		17.0		20.1		14.9	20.0		22.6	24.2

Seaweed (algae)													
No of subjects	11,179		12,961		12,670		6,004		14,596	18,612		17,943	8,175
Rare, %	2.0		1.9		1.8		2.0		1.2	1.3		1.2	1.6
1-2/m, %	12.9		10.7		10.8		8.7		6.1	6.2		6.7	7.1
1-2/w, %	34.8		30.0		29.4		27.4		27.8	24.7		25.0	25.2
3-4/w, %	30.2		30.1		29.4		29.2		34.5	31.2		29.3	28.9
5+/w, %	20.2		27.2		28.6		32.8		30.4	36.6		37.8	37.2

Pickles													
No of subjects	11,162		12,960		12,628		5,912		14,617	18,532		17,848	8,047
Rare, %	4.5		4.7		5.7		7.8		4.6	5.2		5.4	7.0
1-2/m, %	6.1		4.9		5.7		5.2		4.8	4.7		4.8	4.4
1-2/w, %	14.3		12.3		12.3		12.2		12.3	10.9		11.2	11.2
3-4/w, %	19.5		16.3		15.2		14.1		17.4	14.2		13.4	13.0
5+/w, %	55.6		61.8		61.2		60.7		60.9	65.0		65.3	64.3

*Tukudani* (Preserved foods concocted with say souce)													
No of subjects	9,723		11,038		10,663		4,742		12,867	16,046		14,820	6,423
Rare, %	21.5		23.2		23.6		22.4		24.2	27.5		27.9	23.6
1-2/m, %	31.3		28.7		27.6		25.1		29.7	27.9		26.3	24.3
1-2/w, %	28.7		27.5		27.9		28.1		26.9	24.0		24.7	26.8
3-4/w, %	12.9		12.9		14.2		15.2		12.8	12.9		13.4	15.1
5+/w, %	5.7		7.8		6.8		9.2		6.5	7.8		7.7	10.2

Boiled beans													
No of subjects	9,520		10,686		10,458		4,802		12,569	15,768		15,152	6,641
Rare, %	28.7		22.8		17.9		14.2		20.5	15.1		12.6	11.8
1-2/m, %	40.1		38.1		37.2		32.5		45.1	41.0		38.2	32.6
1-2/w, %	21.4		23.9		26.1		28.6		22.0	25.0		26.5	28.0
3-4/w, %	7.4		10.5		13.1		16.3		9.1	13.0		14.8	17.5
5+/w, %	2.4		4.6		5.8		8.4		3.3	6.0		7.9	10.1

Tofu (soybean curd)													
No of subjects	10,272		12,158		12,061		5,670		13,685	17,691		17,221	7,697
Rare, %	1.6		1.3		1.0		1.5		1.0	1.1		1.0	1.7
1-2/m, %	8.1		6.0		6.0		6.8		4.2	4.2		4.4	6.2
1-2/w, %	34.0		30.8		29.5		28.0		28.2	25.7		27.2	28.5
3-4/w, %	35.2		35.3		33.3		32.1		38.4	34.8		32.1	33.7
5+/w, %	21.2		26.6		30.1		31.6		28.1	34.2		35.3	30.0

Citrus fruits													
No of subjects	8,830		10,303		10,395		4,698		11,951	15,522		15,203	6,579
Rare, %	8.1		7.7		7.2		6.5		4.2	4.2		3.3	4.3
1-2/m, %	19.9		16.5		14.8		12.5		11.1	9.8		8.1	7.6
1-2/w, %	28.9		26.2		25.6		24.1		23.6	21.2		19.0	18.2
3-4/w, %	21.6		22.9		23.1		23.9		24.1	23.1		22.3	22.0
5+/w, %	21.5		26.7		29.4		33.1		37.1	41.7		47.3	48.0

Fresh fruit juice (in summer season)													
No of subjects	8,552		9,732		9,492		4,033		11,419	14,420		13,381	5,503
Rare, %	18.3		22.2		24.5		28.7		19.8	23.0		26.8	31.8
1-2/m, %	21.0		17.5		17.5		18.6		18.3	16.5		16.3	15.1
1-2/w, %	29.1		26.0		24.6		22.4		26.2	22.5		21.3	20.0
3-4/w, %	18.1		18.8		17.8		16.0		18.5	18.0		16.4	15.4
5+/w, %	13.6		15.6		15.6		14.2		17.2	20.0		19.3	17.7

Other fruits (excluded citrus fruits)													
No of subjects	8,768		10,107		9,979		4,454		11,783	14,989		14,196	6,120
Rare, %	4.5		5.2		5.4		5.3		2.2	3.2		3.6	3.9
1-2/m, %	15.0		14.4		14.7		13.4		6.8	8.0		8.3	8.7
1-2/w, %	30.3		28.6		27.8		25.6		20.1	20.7		20.2	20.0
3-4/w, %	24.8		24.7		24.4		25.3		27.3	25.2		24.1	24.7
5+/w, %	25.5		27.2		27.7		30.5		43.6	43.0		43.8	42.8

Sweets													
No of subjects	9,801		11,445		11,323		5,173		13,215	16,999		16,264	7,161
Rare, %	22.5		19.4		15.5		11.2		8.4	8.9		9.8	9.8
1-2/m, %	25.4		23.3		21.3		19.4		18.6	19.0		19.5	17.2
1-2/w, %	27.9		27.2		28.1		26.2		30.1	29.5		30.0	28.6
3-4/w, %	15.6		17.5		19.4		21.8		23.5	22.5		21.5	22.3
5+/w, %	8.7		12.6		15.7		21.4		19.5	20.3		19.3	22.2

Coffee													
No of subjects	11,204		13,235		13,115		6,226		14,746	18,946		18,590	8,544
Rare, %	17.3		28.9		34.0		37.8		16.0	29.5		38.3	46.7
1-2/m, %	6.3		8.6		9.7		8.4		7.4	10.2		9.3	8.0
1-2/w, %	16.4		18.4		18.3		19.1		17.6	18.5		16.7	16.5
3-4/w, %	9.9		10.9		10.4		9.7		9.8	9.7		8.4	7.5
5+/w, %	50.1		33.2		27.6		25.0		49.2	32.1		27.4	21.3

Black tea													
No of subjects	9,676		11,102		10,925		4,924		12,778	16,141		15,596	6,803
Rare, %	70.3		76.9		78.5		76.0		64.4	72.8		74.8	75.2
1-2/m, %	16.6		12.7		11.5		11.1		18.5	14.6		12.5	11.1
1-2/w, %	8.1		6.4		6.0		7.1		10.5	7.6		7.3	7.4
3-4/w, %	3.2		2.7		2.6		3.7		4.3	3.4		3.6	3.7
5+/w, %	1.8		1.2		1.4		2.1		2.3	1.6		1.9	2.6

Green tea													
No of subjects	10,925		12,734		12,659		5,991		14,248	18,128		17,680	8,127
Rare, %	8.5		8.7		7.8		9.0		10.0	9.8		9.7	10.0
1-2/m, %	7.7		6.3		5.7		5.7		9.3	7.2		6.7	6.6
1-2/w, %	8.1		5.9		5.1		4.4		7.9	6.2		5.2	4.3
3-4/w, %	18.5		13.4		12.4		13.5		16.0	11.4		11.0	11.5
5+/w, %	57.2		65.8		68.9		67.4		56.9	65.4		67.4	67.7

Oolong tea													
No of subjects	9,190		10,415		10,128		4,503		12,068	15,004		14,346	6,218
Rare, %	67.9		74.9		80.5		83.9		66.1	71.1		75.8	82.2
1-2/m, %	12.2		9.3		6.9		5.0		10.2	8.5		6.9	5.0
1-2/w, %	8.0		5.9		4.6		3.7		7.0	5.9		4.9	3.4
3-4/w, %	5.7		4.4		3.7		3.6		6.4	5.9		4.6	3.2
5+/w, %	6.3		5.5		4.2		3.9		10.3	8.6		7.8	6.2

Japanese style breakfast													
No of subjects	9,724		11,343		11,604		5,566		12,701	16,418		16,612	7,653
Yes, %	81.9		87.3		84.7		82.1		80.3	83.6		80.2	78.9
No, %	18.1		12.8		15.3		17.9		19.7	16.4		19.8	21.1

Westen style breakfast													
No of subjects	9,721		11,343		11,602		5,567		12,702	16,416		16,608	7,653
Yes, %	14.6		10.2		14.0		17.2		21.4	17.1		21.7	21.0
No, %	85.4		89.8		86.1		82.8		78.6	82.9		78.3	79.0

Chagayu (Tea gruel) at breakfast													
No of subjects	9,390		10,876		10,995		5,253		12,206	15,533		15,828	7,249
Yes, %	1.7		3.8		4.5		6.0		2.1	3.3		4.5	6.0
No, %	98.3		96.2		95.5		94.0		97.9	96.7		95.5	94.0

Other style breakfast													
No of subjects	8,624		10,211		10,515		4,986		11,461	15,034		15,282	6,961
Yes, %	1.4		1.7		2.2		2.7		1.3	1.4		2.0	2.1
No, %	98.7		98.3		97.8		97.3		98.8	98.6		98.0	97.9

Non breakfast													
No of subjects	9,725		11,345		11,604		5,567		12,704	16,419		16,614	7,653
Yes, %	6.5		2.8		1.5		1.0		4.4	2.3		1.0	0.8
No, %	93.5		97.3		98.5		99.0		95.7	97.7		99.0	99.2

Bowles of rice (at present)													
No of subjects	11,338		13,354		13,283		6,371		14,819	19,079		18,751	8,723
0/day, %	0.1		0.0		0.1		0.1		0.1	0.1		0.1	0.1
1/day, %	7.1		5.9		8.8		13.2		11.4	8.7		10.8	13.0
2/day, %	18.6		14.4		16.4		18.0		22.7	19.3		21.4	22.2
3/day, %	27.5		28.2		35.0		44.7		44.4	47.4		50.9	53.4
4/day, %	17.3		14.9		12.3		8.5		9.9	10.7		7.3	5.1
5/day, %	15.3		16.5		12.8		7.6		7.3	8.1		5.2	3.3
6/day, %	12.0		16.7		12.6		6.8		3.8	5.3		4.0	2.7
7 and more/day, %	2.2		3.4		2.1		1.1		0.5	0.4		0.4	0.3

Bowles of rice (at 30 years old)													
No of subjects	9,442	11,154		11,532		5,371		12,301		16,333	16,170		7,058
0/day, %	0.0	0.0		0.0		0.0		0.1		0.0	0.0		0.0
1/day, %	3.8	1.2		0.9		1.3		7.3		2.5	1.7		1.9
2/day, %	14.8	8.3		9.3		10.7		18.2		11.3	11.7		13.2
3/day, %	21.2	13.8		12.7		15.2		37.1		23.3	17.3		19.3
4/day, %	15.9	8.9		6.4		6.2		13.0		12.7	10.1		8.2
5/day, %	17.4	14.5		11.7		9.9		12.3		17.3	15.1		11.5
6/day, %	19.2	30.7		32.2		31.5		10.3		27.0	35.0		35.2
7 and more/day, %	7.6	22.6		26.7		25.3		1.8		6.0	9.1		10.6

Frequency of miso soup													
No of subjects	10,946	12,927		12,885		6,211		14,284		18,318	18,127		8,460
almost everyday	71.0	74.8		77.8		77.7		64.7		71.0	72.4		71.2
1/two day, %	16.3	13.0		10.0		8.4		19.1		14.1	11.6		11.3
a few/w, %	9.0	8.5		8.2		8.8		10.1		9.1	9.9		10.0
Rare, %	3.7	3.8		4.1		5.0		6.1		5.8	6.1		7.5

Bowles of miso soup (at present)													
No of subjects	6,848	8,601		9,104		4,278		8,267		11,822	11,957		5,196
1/day, %	27.8	25.5		28.5		34.5		40.0		36.5	39.1		39.8
2/day, %	38.1	36.7		33.6		31.0		39.2		37.3	35.9		33.8
3/day, %	26.9	29.7		32.2		31.2		18.6		23.6	23.0		24.8
4/day, %	3.5	3.7		2.5		1.2		1.3		1.4	1.0		0.7
5/day, %	2.2	2.3		1.8		1.1		0.6		0.7	0.5		0.5
6/day, %	1.4	2.1		1.4		0.9		0.3		0.5	0.5		0.4
7 and more/day, %	0.1	0.2		0.0		0.1		0.1		0.0	0.0		0.0

Bowles of miso soup (at 30 years old)													
No of subjects	6,831	8,579		9,201		4,350		8,376		12,216	12,423		5,285
1/day, %	27.4	19.9		18.8		21.8		38.0		28.2	25.5		25.4
2/day, %	34.8	29.0		24.9		24.0		35.9		30.3	27.5		25.7
3/day, %	28.0	31.0		30.3		30.6		21.5		30.4	32.8		34.8
4/day, %	4.5	6.9		6.9		5.8		2.4		4.4	4.8		4.6
5/day, %	2.8	5.7		7.9		6.5		1.2		3.5	4.4		3.9
6/day, %	2.1	6.7		10.2		10.3		0.9		3.1	4.7		5.3
7 and more/day, %	0.4	0.8		1.0		1.1		0.1		0.2	0.3		0.3

Taste for salty foods													
No of subjects	9,728	11,314		11,310		5,275		12,793		16,183	15,935		7,066
love, %	14.1	13.1		9.6		8.2		7.3		6.4	5.2		6.5
like, %	34.2	31.5		28.3		25.0		21.6		20.1	19.5		19.6
soso, %	41.5	43.7		49.0		50.5		54.1		55.2	55.9		52.6
not like, %	8.9	9.9		11.0		13.2		15.2		15.9	16.4		17.0
hate, %	1.3	1.9		2.1		3.1		1.8		2.5	3.0		4.3

Change of taste for salty foods													
No of subjects	9,394	10,938		11,069		5,241		12,289		15,765		15,824	6,987
decreased, %	33.9	54.5		69.0		75.4		39.5		64.8		77.4	77.1
stable, %	64.2	44.2		30.0		23.6		57.9		34.0		21.8	21.8
increased, %	2.0	1.3		1.0		1.1		2.6		1.2		0.9	1.1

Taste for fatty foods													
No of subjects	9,715	11,315		11,424		5,390		12,796		16,293		16,250	7,333
love, %	8.2	7.3		5.6		5.2		3.3		2.4		2.0	1.9
like, %	26.3	22.7		22.5		20.3		16.3		12.0		10.3	9.8
soso, %	45.7	47.6		49.5		50.0		53.8		53.3		51.1	46.4
not like, %	17.7	19.7		19.8		20.9		23.8		28.4		31.8	33.9
hate, %	2.1	2.7		2.6		3.7		2.9		3.8		4.9	8.1

Change of taste for fatty foods													
No of subjects	9,387	10,809		10,928		5,144		12,243		15,566		15,509	6,874
decreased, %	36.0	49.5		59.3		65.0		46.4		62.0		71.0	70.5
stable, %	62.9	49.3		38.7		32.7		52.7		36.8		27.2	27.3
increased, %	1.2	1.3		2.0		2.4		1.0		1.2		1.8	2.2

Modification of salt intake													
No of subjects	9,289	10,897		11,035		5,170		12,020		15,744		15,494	7,030
Yes, %	25.1	36.9		46.1		52.9		28.7		44.0		52.9	51.9
No, %	74.9	63.1		53.9		47.1		71.3		56.0		47.1	48.1

Modification of sugar intake													
No of subjects	9,289	10,894		11,029		5,171		12,017		15,741		15,484	7,024
Yes, %	13.6	18.6		21.5		26.1		18.3		26.3		30.6	28.8
No, %	86.4	81.4		78.5		73.9		81.7		73.7		69.4	71.2

Modification of energy intake													
No of subjects	9,290	10,894		11,024		5,171		12,015		15,738		15,480	7,019
Yes, %	6.0	8.4		9.7		10.9		7.2		10.5		12.2	11.3
No, %	94.0	91.6		90.3		89.1		92.8		89.5		87.8	88.7

Modification of fat intake													
No of subjects	9,288	10,897		11,028		5,171		12,021		15,739		15,479	7,025
Yes, %	14.6	18.1		21.6		25.1		17.8		26.6		31.4	30.3
No, %	85.4	81.9		78.5		74.9		82.2		73.4		68.6	69.7

### Meat and liver

For both men and women, the percentage of individuals reporting intake of 3+ times/week was about 5 to 15% for beef, 15 to 25% for pork (excluding ham and sausages), 15 to 20% for ham and chicken and 5% for liver. Only in men the percentage reporting intake 3+ times/week for beef and chicken increased with age while in both sexes the percentage decreased with age for pork and ham. The percentage of high intake was similar among age groups for liver in men and women and for beef and chicken in women.

### Eggs and dairy products

The percentage of individuals with intake 3+ times/week was about 70% for eggs, 50 to 60% for milk, 5 to 15% for yogurt, 5 to 10% for cheese, and 10% for butter, 15 to 30% for margarine. Women reported the highest frequency of margarine than men, but the frequency was similar in both sexes for other foods. The percentage of intake 3+ times/week increased with age for milk, yogurt, cheese, butter and margarine in men while the percentage decreased with age for margarine in women. The percentage of high intake was similar among the age groups for eggs in men and women and for milk, yogurt, cheese and butter in women. Egg intake at 5+ times/week was reported by 35 to 50% of individuals, and the frequency increased with age in men but not in women.

### Deep-fried foods, and fried vegetables

The percentage of individuals with intake 3+ times/week was about 20 to 25% for deep-fried foods or tempura, and 25 to 40% for fried vegetables, in both men and women. In general, vegetable oils were used for frying. The percentage of individuals with intake 3+ times/week increased with age for fried vegetables while it did not change with age for deep-fried foods in both sexes.

### Raw fish and fish products

The percentage of individuals reporting intake 3+ times/week was about 55 to 65% for raw fish, 10 to 15% for Kamaboko (fish paste), and 25 to 30% for Himono (dried fish or salted fish). A higher frequency of fresh fish intake was reported by women than men, but the frequency was similar for fish products. The percentage of individuals with intake 5+ times/week increased with age for fish products but not for fresh fish. The percentage of raw fish intake 5+ times/week was 20 to 30%, and increased with age in men but not in women. Only 10% of men and women had raw fish intake less than 1 time/week.

### Vegetables, fungi, seaweed and beans

The percentage of individuals with intake 5+ times/week (almost every day) was about 20 to 40% for Spinach or garland chrysanthemumm, 10 to 25% for carrot or pumpkin, 5 to 20% for tomatoes, 20 to 30% for cabbage or head lettuce, 10 to 20% for Chinese cabbage, 5% for Sansai (edible wild plants), 5 to 10% for fungi (enokidake, shiitake, mushroom), 10 to 25% for potatoes, 20 to 40% for seaweed (algae), 55 to 65% for pickles, 5 to 10% for Tukudani (Preserved foods concocted with say sauce), 3 to 10% for boiled beans, and 20 to 35% for Tofu (soybean curd). Women reported a higher frequency of these food intakes than men. The percentage of individuals with intake 5+ times/week increased with age for all of these food items.

### Fruits

The percentage of individuals with intake 5+ times/week (almost every day) was about 20 to 50% for citrus fruits, 10 to 20% for fresh fruit juice, 25 to 45% for other fruits (excluding citrus fruit). The frequency of intakes of fruits was higher in women than men. The percentage of individuals with intake 5+ times/week increased with age for citrus fruits in men and women, and for other fruits in men. The percentage of the higher intake was similar among the age groups for fresh fruit juice in men and women and for other fruits in women.

### Sweets and beverages

The percentage of individuals with intake 5+ times/week (almost every day) was about 10 to 20% for sweets, 20 to 50% for coffee, 1 to 3% for black tea, 55-70% for green tea, 3 to 10% for oolong tea. The frequency of intake of sweets and oolong tea was high in women than men, but the frequency was similar in both sexes for coffee, black tea and green tea. The percentage of individuals with intake 5+ times/week increased with age for sweets, green tea, and decreased with age for coffee and oolong tea in both sexes.

### Type of breakfast

The percentage of individuals who reported having Japanese style breakfast was 80%, western style breakfast 10 to 20%, Chagayu (Tea gruel) 5%, other types 2% and no breakfast less than 5%. Having western style breakfast was highest in women but the frequency was similar in both sexes for Japanese style, Chagayu, other styles or no breakfast.

### Rice

The percentage of individuals who reported eating rice at 3+ bowls/day was 65 to 80% at present and 80 to 90% at age of 30 years. More men reported eating rice than women. The percentage of individuals who reported eating rice at a frequency of 6+ bowls/day was 5 to 20% at present and 10 to 60% at age of 30 years. The percentage of rice intake 3+ bowls/day at present decreased with age in men and women while that at age of 30 years increased with age in both sexes.

### Miso soup

The percentage of individuals who reported consuming miso soup (soy bean soup) every day was 65 to 80% at present with a higher frequency of men than women. The percentage of individuals who consumed 3+ bowls of miso soup/day was 20 to 40% at present and 25 to 60% at age of 30 years with higher frequency of men than women. The percentage of individuals who consumed 6+ bowls miso soup/day at present was less than 2% of men and women while that at age of 30 years was 3 to 10% of men and 1 to 5% of women. The proportion of individuals reporting consuming miso soup increased with age in both sexes.

### Taste for salty and fatty foods

Thirty to 50% of individuals reported preference of salty foods, with a higher frequency among men than women. This percentage decreased with age in both sexes. On the other hand, 30 to 75% of individuals reported preference of low-salt food, with a higher frequency among women than men. This percentage increased substantially with age in both sexes.

Preference of fatty food was reported by 10 to 35% of individuals with a higher frequency among men than women. This percentage decreased with age in both sexes. On the other hand, the percentage of individuals preferring low-fat food was 35 to 70% with a higher frequency among women than men. This percentage increased substantially with age in both sexes.

### Modification for salt, sugar, energy and fat

Twenty-five to 55% of individuals reported desire for modification of food with respect to salt, 15 to 30% to sugar, 5 to 15% to energy, and 15 to 30% to fat in both men and women. These percentages increased with age in both sexes.

### Estimated nutrient and food intake

Table 2 shows the estimated major nutrient intake according to sex and age. The mean total energy was about 1500 to 1700 kcal/day in men and 1250 to 1300 kcal/day in women. The mean total energy was highest in ages 50-59 and lowest in ages 70-79 for both sexes.

**Table 2.** Nutrient intake (mean and standard deviation) according to sex and age.

		Men				Women			

		Age (years)				Age (year)			

		40-49	50-59	60-69	70-79	40-49	50-59	60-69	70-79
No. of subjects		7,091	7,652	6,928	2,715	10,387	12,296	10,651	4,159
Total weight	g/day	1692.46 ± 495.18	1791.01 ± 516.32	1738.53 ± 508.35	1629.41 ± 497.2	1658.05 ± 445.57	1696.54 ± 453.93	1645.07 ± 457.18	1567.55 ± 458.2
Total enargy	kcal/day	1636.25 ± 464.72	1723.11 ± 474.33	1611 ± 465.16	1462.7 ± 422.6	1329.46 ± 344.6	1359.02 ± 346.95	1309.31 ± 335.31	1242.74 ± 322.55
Water	g/day	1375.48 ± 428.46	1450.84 ± 451.77	1414.97 ± 445.47	1328.39 ± 439.0	1361.12 ± 397.99	1390.26 ± 408.22	1348.98 ± 410.78	1285.11 ± 413.62
Animal protein	g/day	24.5 ± 9.6	26.4 ± 10.1	26.0 ± 10.1	25.1 ± 10.1	26.5 ± 9.7	26.7 ± 9.9	25.5 ± 10.0	23.8 ± 9.9
Vegetable protein	g/day	27.5 ± 9.3	29.7 ± 9.6	28.7 ± 9.3	27.1 ± 8.7	25.2 ± 7.5	26.6 ± 7.6	26.3 ± 7.5	25.4 ± 7.4
Total protein	g/day	51.9 ± 15.9	56.1 ± 16.5	54.7 ± 16.2	52.2 ± 15.7	51.7 ± 14.6	53.4 ± 14.9	51.8 ± 15.0	49.2 ± 14.8
Animal fat	g/day	13.2 ± 5.7	13.4 ± 5.7	13.2 ± 5.7	13.0 ± 5.8	14.7 ± 6.0	13.8 ± 5.8	13.1 ± 5.8	12.0 ± 5.7
Vegetable fat	g/day	13.6 ± 5.2	14.4 ± 5.6	14.5 ± 5.4	14.1 ± 5.3	13.8 ± 4.9	14.1 ± 5.1	14.0 ± 5.1	13.2 ± 5.1
Total fat	g/day	30.1 ± 10.4	31.6 ± 10.8	31.4 ± 10.7	30.6 ± 10.7	31.9 ± 10.4	31.7 ± 10.5	30.8 ± 10.6	28.7 ± 10.5
Carbohydrate	g/day	220.3 ± 75.5	236.6 ± 77.7	221.6 ± 75.3	202.8 ± 68.2	198.5 ± 58.1	205.7 ± 57.7	198.1 ± 55.0	189.7 ± 52.6
Crude fiber	g/day	2.8 ± 1.1	3.0 ± 1.1	3.1 ± 1.1	3.0 ± 1.1	2.8 ± 0.9	3.0 ± 1.0	3.1 ± 1.0	3.0 ± 1.0
Ash	g/day	11.4 ± 4.2	12.2 ± 4.4	12.2 ± 4.2	11.9 ± 4.1	11.4 ± 3.7	11.9 ± 3.9	11.8 ± 4.0	11.4 ± 4.0
Calcium	mg/day	435.2 ± 161.3	462.1 ± 162.9	465.0 ± 158.8	460.0 ± 158.9	459.9 ± 156.9	472.6 ± 155.7	469.5 ± 154.8	444.0 ± 155.9
Phosphate	mg/day	734.1 ± 225.2	788.7 ± 229.6	773.7 ± 226.9	745.4 ± 222.7	743.0 ± 211.1	764.3 ± 213.1	745.8 ± 214.2	708.0 ± 212.4
Iron	mg/day	7.5 ± 2.7	8.2 ± 2.9	8.3 ± 2.9	8.1 ± 2.8	7.5 ± 2.5	8.0 ± 2.6	8.0 ± 2.7	7.8 ± 2.7
Sodium	mg/day	2095 ± 963	2246 ± 1029	2236 ± 974	2157 ± 936	1977 ± 841	2086 ± 885	2069 ± 898	2010 ± 890
Potassium	mg/day	1847 ± 602	1983 ± 629	1990 ± 626	1965 ± 632	1968 ± 565	2050 ± 589	2037 ± 601	1959 ± 616
Retinol	µg/day	536 ± 721	613 ± 873	609 ± 885	609 ± 927	545 ± 805	554 ± 866	542 ± 880	494 ± 875
Carotin	µg/day	1686 ± 841	1915 ± 908	2004 ± 916	2050 ± 931	1934 ± 837	2131 ± 886	2197 ± 909	2126 ± 923
Vitamin A	IU/day	2739 ± 2528	3120 ± 3042	3156 ± 3082	3180 ± 3216	2909 ± 2802	3046 ± 3004	3041 ± 3059	2838 ± 3041
Vitamin B_1_	mg/day	0.75 ± 0.25	0.80 ± 0.26	0.78 ± 0.25	0.76 ± 0.25	0.77 ± 0.23	0.79 ± 0.23	0.77 ± 0.24	0.73 ± 0.23
Vitamin B_2_	mg/day	0.98 ± 0.34	1.06 ± 0.36	1.05 ± 0.36	1.03 ± 0.37	1.04 ± 0.35	1.06 ± 0.36	1.04 ± 0.36	0.98 ± 0.36
Niacine	mg/day	10.5 ± 3.5	11.4 ± 3.7	11.1 ± 3.7	10.6 ± 3.7	10.7 ± 3.3	11.1 ± 3.5	10.7 ± 3.6	10.2 ± 3.5
Vitamin C	mg/day	91.5 ± 39.5	100.7 ± 41.1	103.4 ± 40.8	102.8 ± 41.5	105.6 ± 37.6	112.2 ± 39.5	113.3 ± 39.7	109.1 ± 40.9
Salt	g/day	5.19 ± 2.42	5.57 ± 2.59	5.55 ± 2.45	5.35 ± 2.35	4.89 ± 2.12	5.17 ± 2.22	5.13 ± 2.26	4.98 ± 2.24
Cholesterol	mg/day	226.4 ± 91.6	249.1 ± 95.6	246.6 ± 95.4	239.2 ± 97.1	243.0 ± 91.7	244.7 ± 94.8	235.1 ± 96.5	224.7 ± 95.6
*α*-tocopherol	mg/day	3.70 ± 1.18	4.09 ± 1.22	4.04 ± 1.21	3.89 ± 1.19	3.79 ± 1.06	4.02 ± 1.10	3.97 ± 1.13	3.80 ± 1.12
*β*-tocopherol	mg/day	0.12 ± 0.06	0.13 ± 0.07	0.13 ± 0.06	0.13 ± 0.06	0.12 ± 0.06	0.12 ± 0.06	0.12 ± 0.06	0.12 ± 0.06
*γ*-tocopherol	mg/day	3.96 ± 2.14	4.27 ± 2.26	4.31 ± 2.15	4.19 ± 2.08	3.66 ± 1.84	3.96 ± 1.92	3.98 ± 1.94	3.86 ± 1.97
*δ*-tocopherol	mg/day	1.59 ± 0.95	1.72 ± 1.00	1.73 ± 0.96	1.68 ± 0.93	1.40 ± 0.81	1.54 ± 0.85	1.56 ± 0.86	1.53 ± 0.87
Vitamin E	mg/day	3.93 ± 1.27	4.33 ± 1.32	4.28 ± 1.30	4.13 ± 1.27	3.99 ± 1.13	4.24 ± 1.18	4.20 ± 1.20	4.02 ± 1.19
Total fatty acids	mg/day	26.0 ± 8.9	27.3 ± 9.2	27.0 ± 9.1	26.3 ± 9.1	27.1 ± 8.8	27.1 ± 8.9	26.2 ± 8.9	24.5 ± 8.8
Saturted fatty acids	mg/day	9.05 ± 3.35	9.29 ± 3.25	9.12 ± 3.26	8.87 ± 3.28	9.62 ± 3.39	9.31 ± 3.25	8.93 ± 3.23	8.21 ± 3.17
Monounsaturted fatty acids	mg/day	9.11 ± 3.31	9.63 ± 3.48	9.54 ± 3.44	9.30 ± 3.47	9.76 ± 3.37	9.69 ± 3.41	9.32 ± 3.44	8.70 ± 3.38
Polyunsaturted fatty acids	mg/day	7.64 ± 2.88	8.31 ± 3.09	8.26 ± 3.00	8.01 ± 2.91	7.59 ± 2.66	7.96 ± 2.79	7.82 ± 2.83	7.49 ± 2.79
n3-fatty acids	mg/day	1.53 ± 0.66	1.70 ± 0.70	1.70 ± 0.70	1.64 ± 0.68	1.58 ± 0.64	1.70 ± 0.67	1.65 ± 0.68	1.57 ± 0.68
n6-fatty acids	mg/day	6.11 ± 2.31	6.61 ± 2.49	6.55 ± 2.41	6.36 ± 2.33	6.01 ± 2.12	6.26 ± 2.22	6.16 ± 2.25	5.91 ± 2.21

The mean values of intake of animal and vegetable proteins were approximately 25 g/day in men and women of all age groups. The mean values of intake of animal and vegetable fats were about 12 to 15 g/day in men and women. The mean animal fat intake was lower in ages 70-79 than in other age groups for women, but the mean vegetable fat intake was not among the different age groups.

The mean carbohydrate intake was 190 to 200 g/day in men and women with higher intake by men than women. The mean carbohydrate intake was highest in ages 50-59 and lowest in ages 70-79 for both sexes.

The mean crude fiber intake was about 3 g/day for both sexes, and it was lower in ages of 40-49 than in other age groups. The mean calcium intake was around 450 mg/day, and it was lower in men aged 40-49 and women aged 70-79. The mean phosphate intake was about 700 to 800 mg/day in men and women. The mean phosphate intake was highest in ages 50-59 than in other age groups.

The mean iron intake was about 7.5 to 8.5 g/day for both sexes, and it was lower in ages of 40-49 than in other age groups. The mean sodium intake was around 2,000 mg/day with a higher intake in men than in women. The mean sodium intake was higher in ages 50-69 than in other age groups. The mean potassium intake was 1,800 to 2,000 mg/day with a higher intake in women than men for all age groups except for ages 70-79. The mean potassium intake was higher in ages 50-69 than in other age groups.

The mean retinol intake was about 500 to 600 mg/day with a higher intake in men than in women. The mean retinol intake was lower in men aged 40-49 and in women aged 70-79 than in other sex and age groups. The mean carotene intake was 1,700 to 2,000 mg/day in men and 1,900 to 2,200 mg/day in women, and it increased with age in both sexes except for women aged 70-79. The mean vitamin A intake was 2,700 to 3,200 U/day in both men and women, and it increased with age except for women aged 70-79.

The mean values of vitamins B_1_, B_2_ and niacin intake were similar between men and women, and among age groups. The mean vitamin C intake was about 90 to 100 mg/day in men and 100 to 110 mg/day in women. The intake was lower in ages of 40-49 than in other age groups in men and women.

The mean cholesterol intake was 230 to 250 mg/day in both men and women, and it was lower in men aged 40-49 and women aged 70-79 than in other sex and age groups. The mean values of intake of alpha to gamma- tocopherol and vitamin E were similar between the sexes, and it was lowest in ages of 40-49 for both sexes.

The mean saturated fat intake was about 9 g/day and mean monounsaturated fat was 9 to 10 g/day in both men and women, and they were lower in men aged 70-79 and women aged 60-79 than other sex and age groups. The mean polyunsaturated fat intake was about 8 g/day in both sexes, and it was lowest in men aged 40-49 than men of other age groups. The mean intake of n3 fatty acids was about 2 g/day and the mean intake of n6 fatty acids was about 6 g/day in both sexes of all age groups.

### Analyses of food and nutrient intake according to sex and geographical parameters

Table 3 shows the frequency of food intake according to sex by geographical area. The percentages of men and women who consumed more meats, eggs, deep-fried foods, fried vegetables, and fresh fish were generally higher in Tohoku and Kyushu than in other areas. The percentages of men and women who consumed more milk and dairy products were higher in Hokkaido and Chubu than in other areas. The percentage of individuals who consumed more vegetables was higher in Tohoku and Chubu and lower in Kinki and Chugoku. The percentage of individuals who consumed more green tea was lower in Hokkaido. The percentage of individuals who consumed more rice was higher in Hokkaido, Tohoku and Kinki, and lower in Chugoku. The percentage of individuals who consumed more miso soup was higher in Hokkaido and Tohoku and lower in Kinki and Kyushu. The percentage of individuals who preferred salty foods was lower in Hokkaido and Kyushu than in other areas. The percentage of individuals who preferred fatty foods was lower in Kyushu than in other areas.

**Table 3.** Sex-specific age-adjusted percentages of higher frequency of foods by geographical area.

	Hokkaido	Tohoku	Kanto	Chubu	Kinki	Chugoku	Kyusyu
						
	No. ( % )	No. ( % )	No. ( % )	No. ( % )	No. ( % )	No. ( % )	No. ( % )
Men							
3-4/w and more, %							
Beef	1,030 ( 2.9 )	3,782 ( 5.0 )	4,909 ( 5.6 )	9,252 ( 6.3 )	7,656 ( 15.8 )	4,581 ( 10.7 )	1,136 ( 15.8 )
Pork (excluding ham and sausages)	1,459 ( 25.0 )	5,648 ( 27.2 )	4,987 ( 25.7 )	9,863 ( 28.0 )	7,222 ( 10.9 )	4,218 ( 9.1 )	1,117 ( 13.8 )
Ham	1,448 ( 15.5 )	5,187 ( 17.7 )	7,866 ( 21.3 )	8,909 ( 16.8 )	7,037 ( 10.8 )	3,922 ( 12.0 )	3,637 ( 26.6 )
Chiken	1,453 ( 16.9 )	5,506 ( 23.9 )	7,982 ( 20.1 )	9,409 ( 16.4 )	7,339 ( 18.9 )	4,274 ( 10.0 )	1,106 ( 20.4 )
Liver	1,436 ( 1.8 )	3,725 ( 7.9 )	4,066 ( 3.3 )	8,743 ( 3.8 )	6,356 ( 3.7 )	4,019 ( 2.7 )	5,172 ( 8.0 )
Eggs	1,459 ( 69.7 )	5,875 ( 71.5 )	8,110 ( 70.9 )	10,341 ( 74.2 )	7,683 ( 66.1 )	4,730 ( 65.1 )	5,412 ( 80.5 )
Milk	1,456 ( 59.7 )	5,481 ( 49.9 )	8,005 ( 53.6 )	9,896 ( 65.1 )	7,308 ( 46.7 )	4,427 ( 55.5 )	5,336 ( 52.3 )
Yogurt	357 ( 8.7 )	3,536 ( 7.2 )	7,709 ( 7.9 )	8,613 ( 10.7 )	6,794 ( 4.3 )	3,830 ( 8.6 )	1,090 ( 8.0 )
Cheese	1,451 ( 8.6 )	5,016 ( 5.2 )	7,025 ( 7.7 )	8,715 ( 8.9 )	5,921 ( 3.9 )	3,825 ( 6.2 )	1,098 ( 3.6 )
Butter	1,451 ( 12.9 )	5,019 ( 6.6 )	6,989 ( 10.4 )	8,549 ( 8.1 )	5,902 ( 8.5 )	3,775 ( 11.5 )	1,090 ( 5.8 )
Margarine	1,026 ( 15.2 )	5,068 ( 11.7 )	4,047 ( 10.0 )	8,587 ( 15.6 )	5,883 ( 13.8 )	3,827 ( 23.3 )	5,076 ( 20.9 )
Deep-fried foods or *tempura*	1,453 ( 18.0 )	5,561 ( 33.2 )	4,187 ( 22.9 )	9,683 ( 23.8 )	6,338 ( 20.0 )	4,379 ( 14.3 )	1,128 ( 26.6 )
Fried vegetables	1,450 ( 45.8 )	5,453 ( 49.0 )	4,233 ( 35.0 )	10,019 ( 31.7 )	6,463 ( 27.9 )	4,530 ( 29.1 )	1,128 ( 38.1 )
Fresh fish	1,460 ( 58.1 )	5,818 ( 69.0 )	5,043 ( 49.9 )	10,264 ( 56.8 )	7,651 ( 51.7 )	4,778 ( 50.6 )	5,315 ( 76.6 )
*Kamaboko* (fish paste)	244 ( 14.7 )	3,646 ( 18.9 )	7,887 ( 15.4 )	8,899 ( 8.5 )	6,203 ( 12.8 )	4,236 ( 14.5 )	1,119 ( 18.2 )
*Himono* (Dried fish or salted fish)	1,454 ( 26.5 )	5,501 ( 37.5 )	4,946 ( 24.2 )	9,591 ( 27.8 )	7,460 ( 33.9 )	4,318 ( 17.7 )	1,128 ( 15.7 )

5/w and more, %							
Spinach or garland chrysanthemumm	1,037 ( 23.6 )	5,852 ( 38.2 )	7,325 ( 32.4 )	10,110 ( 27.3 )	6,963 ( 24.8 )	4,667 ( 29.4 )	1,139 ( 26.7 )
Carrot or pumpkin	1,457 ( 13.9 )	5,616 ( 17.9 )	5,004 ( 13.8 )	9,921 ( 19.2 )	7,034 ( 7.9 )	4,378 ( 7.3 )	1,132 ( 12.1 )
Tomatoes	1,462 ( 11.1 )	5,566 ( 18.3 )	7,858 ( 12.0 )	9,302 ( 8.6 )	7,147 ( 9.9 )	4,294 ( 6.3 )	1,122 ( 17.8 )
Cabbage or head lettuce	1,458 ( 26.0 )	5,677 ( 27.8 )	5,012 ( 22.5 )	9,992 ( 26.1 )	6,790 ( 14.8 )	4,598 ( 20.6 )	1,128 ( 17.8 )
Chinese cabbage	909 ( 8.8 )	5,538 ( 20.1 )	4,191 ( 17.4 )	9,929 ( 23.5 )	6,379 ( 10.3 )	4,173 ( 7.5 )	1,107 ( 7.3 )
*Sansai* (Edible wild plants)	1,449 ( 1.0 )	5,472 ( 7.0 )	7,060 ( 2.0 )	9,069 ( 1.4 )	7,053 ( 1.9 )	4,134 ( 1.7 )	1,106 ( 1.3 )
Fungi (enokidake, shiitake, mushroom)	927 ( 2.5 )	5,605 ( 8.7 )	4,196 ( 4.6 )	9,668 ( 8.8 )	6,684 ( 4.5 )	4,480 ( 5.3 )	1,116 ( 4.1 )
Potatoes	1,460 ( 9.8 )	5,658 ( 15.3 )	8,035 ( 10.6 )	9,886 ( 33.0 )	7,480 ( 6.8 )	4,466 ( 4.4 )	5,384 ( 8.6 )
Seaweed (algae)	1,463 ( 15.3 )	5,747 ( 28.4 )	8,101 ( 29.1 )	9,937 ( 33.2 )	7,585 ( 22.2 )	4,600 ( 21.9 )	5,381 ( 21.7 )
Pickles	1,454 ( 54.5 )	5,698 ( 55.8 )	8,081 ( 63.6 )	9,941 ( 64.1 )	7,567 ( 59.5 )	4,556 ( 48.9 )	5,365 ( 62.1 )
*Tukudani* (Preserved foods concocted with say souce)	1,457 ( 2.4 )	5,250 ( 7.0 )	7,958 ( 8.1 )	9,033 ( 6.1 )	7,172 ( 9.5 )	4,185 ( 5.8 )	1,111 ( 4.6 )
Boiled beans	1,451 ( 1.5 )	5,297 ( 4.9 )	7,183 ( 5.4 )	9,543 ( 6.5 )	6,518 ( 4.2 )	4,371 ( 3.3 )	1,103 ( 1.6 )
Tofu (soybean curd)	1,463 ( 23.4 )	5,881 ( 45.2 )	5,054 ( 23.1 )	10,308 ( 26.4 )	7,317 ( 20.1 )	4,750 ( 24.3 )	5,388 ( 24.6 )
Citrus fruits	1,451 ( 8.3 )	5,526 ( 15.6 )	4,993 ( 30.8 )	9,911 ( 38.3 )	6,759 ( 27.3 )	4,466 ( 20.8 )	1,120 ( 14.7 )
Fresh fruit juice (in summer season)	1,449 ( 8.4 )	5,299 ( 15.3 )	4,102 ( 14.2 )	9,156 ( 16.1 )	6,511 ( 16.7 )	4,195 ( 14.4 )	1,097 ( 5.2 )
Other fruits (excluded citrus fruits)	1,448 ( 20.7 )	5,317 ( 24.6 )	4,867 ( 22.9 )	9,325 ( 42.4 )	7,068 ( 17.2 )	4,199 ( 24.5 )	1,084 ( 16.6 )
Sweets	1,459 ( 10.7 )	5,626 ( 14.8 )	4,980 ( 16.6 )	9,932 ( 12.9 )	7,493 ( 10.3 )	4,583 ( 13.8 )	3,669 ( 18.5 )
Coffee	1,461 ( 12.9 )	5,904 ( 18.6 )	8,262 ( 15.5 )	10,475 ( 90.3 )	7,793 ( 41.6 )	4,983 ( 60.6 )	4,902 ( 5.3 )
Black tea	1,032 ( 0.1 )	5,658 ( 0.9 )	7,229 ( 0.9 )	9,597 ( 2.0 )	7,375 ( 1.6 )	4,617 ( 2.9 )	1,119 ( 0.5 )
Green tea	1,458 ( 23.1 )	5,960 ( 55.9 )	7,493 ( 72.8 )	10,415 ( 73.1 )	7,732 ( 74.9 )	4,810 ( 48.7 )	4,441 ( 42.9 )
Oolong tea	922 ( 2.0 )	5,046 ( 3.7 )	7,063 ( 2.4 )	9,341 ( 8.1 )	6,325 ( 3.3 )	4,449 ( 7.4 )	1,090 ( 6.8 )

3/day and more, %							
Bowles of rice (at present)	1,459 ( 84.4 )	6,111 ( 82.7 )	8,310 ( 76.0 )	10,411 ( 74.8 )	7,752 ( 80.3 )	4,965 ( 59.8 )	5,310 ( 71.5 )
Bowles of rice (30 years old)	1,418 ( 91.1 )	5,799 ( 93.5 )	7,879 ( 85.9 )	9,658 ( 85.8 )	7,020 ( 90.6 )	4,620 ( 80.5 )	1,094 ( 91.2 )
Bowles of miso soup (at present)	1,307 ( 62.2 )	5,810 ( 63.3 )	6,221 ( 29.0 )	8,364 ( 36.7 )	3,505 ( 12.7 )	2,885 ( 20.6 )	742 ( 16.0 )
Bowles of miso soup (30 years old)	1,337 ( 68.7 )	5,467 ( 78.5 )	6,275 ( 45.3 )	8,168 ( 56.1 )	3,806 ( 20.6 )	3,161 ( 28.1 )	747 ( 26.2 )

love or like, %							
Taste for salty food	1,462 ( 33.3 )	4,223 ( 40.0 )	8,096 ( 45.1 )	10,147 ( 43.1 )	7,744 ( 41.9 )	4,840 ( 42.6 )	1,115 ( 24.9 )
Taste for fatty food	1,461 ( 29.8 )	4,254 ( 24.8 )	8,092 ( 31.7 )	10,234 ( 35.1 )	7,780 ( 27.1 )	4,900 ( 28.5 )	1,123 ( 15.1 )

Women							
3-4/w and more, %							
Beef	1,581 ( 1.6 )	5,116 ( 3.4 )	6,149 ( 4.9 )	10,242 ( 5.0 )	9,672 ( 16.6 )	8,418 ( 14.1 )	4,009 ( 19.4 )
Pork (excluding ham and sausages)	2,246 ( 26.9 )	7,820 ( 24.8 )	6,251 ( 28.7 )	11,124 ( 27.9 )	9,210 ( 13.7 )	7,620 ( 11.5 )	3,959 ( 18.4 )
Ham	2,230 ( 14.3 )	7,336 ( 16.9 )	9,493 ( 23.2 )	10,115 ( 17.0 )	8,992 ( 11.4 )	7,123 ( 11.1 )	7,087 ( 20.3 )
Chiken	2,246 ( 20.7 )	7,839 ( 25.5 )	9,652 ( 24.4 )	10,809 ( 18.8 )	9,386 ( 23.7 )	7,878 ( 13.7 )	3,948 ( 26.8 )
Liver	2,219 ( 1.5 )	5,167 ( 5.6 )	4,961 ( 3.2 )	9,785 ( 3.4 )	8,290 ( 3.8 )	7,434 ( 2.9 )	9,013 ( 7.8 )
Eggs	2,251 ( 69.8 )	8,262 ( 69.7 )	9,751 ( 73.0 )	11,863 ( 73.4 )	9,763 ( 66.1 )	8,776 ( 63.8 )	9,425 ( 76.8 )
Milk	2,243 ( 66.3 )	7,856 ( 53.6 )	9,625 ( 61.0 )	11,479 ( 68.3 )	9,483 ( 54.6 )	8,352 ( 63.9 )	9,319 ( 60.3 )
Yogurt	519 ( 10.8 )	5,005 ( 11.6 )	9,324 ( 13.9 )	9,933 ( 16.1 )	8,872 ( 7.5 )	7,261 ( 13.8 )	3,940 ( 13.5 )
Cheese	2,230 ( 8.4 )	7,176 ( 6.2 )	8,190 ( 9.0 )	9,870 ( 10.1 )	7,588 ( 4.1 )	7,088 ( 6.9 )	3,960 ( 5.5 )
Butter	2,228 ( 15.1 )	7,202 ( 8.3 )	8,173 ( 13.3 )	9,631 ( 9.8 )	7,561 ( 10.0 )	6,908 ( 13.7 )	3,927 ( 7.6 )
Margarine	1,565 ( 22.7 )	7,256 ( 13.5 )	4,905 ( 15.9 )	9,739 ( 20.7 )	7,547 ( 22.7 )	7,218 ( 38.1 )	8,923 ( 27.6 )
Deep-fried foods or tempura	2,235 ( 17.2 )	7,888 ( 30.2 )	5,133 ( 21.6 )	11,212 ( 23.6 )	8,085 ( 19.5 )	8,199 ( 13.5 )	3,983 ( 24.4 )
Fried vegetables	2,245 ( 55.8 )	7,859 ( 52.2 )	5,174 ( 39.4 )	11,685 ( 37.4 )	8,178 ( 30.3 )	8,540 ( 29.7 )	4,004 ( 40.9 )
Raw fish	2,250 ( 65.8 )	8,183 ( 72.4 )	6,344 ( 50.8 )	11,697 ( 58.4 )	9,668 ( 52.7 )	8,735 ( 51.9 )	9,201 ( 74.2 )
Kamaboko (fish paste)	405 ( 14.8 )	5,070 ( 20.0 )	9,512 ( 17.9 )	10,150 ( 10.5 )	7,874 ( 14.3 )	7,839 ( 14.0 )	3,974 ( 20.2 )
Himono (Dried fish or salted fish)	2,234 ( 29.2 )	7,827 ( 37.5 )	6,227 ( 25.9 )	10,880 ( 29.5 )	9,477 ( 34.9 )	7,725 ( 15.8 )	3,981 ( 19.3 )

5/w and more, %							
Spinach or garland chrysanthemumm	1,598 ( 28.9 )	8,255 ( 43.6 )	8,613 ( 36.9 )	11,607 ( 30.0 )	8,976 ( 30.2 )	8,605 ( 37.6 )	4,029 ( 35.8 )
Carrot or pumpkin	2,247 ( 22.9 )	8,071 ( 24.8 )	6,368 ( 24.5 )	11,659 ( 28.9 )	9,009 ( 15.4 )	8,309 ( 14.8 )	4,038 ( 25.4 )
Tomatoes	2,241 ( 20.6 )	7,903 ( 26.4 )	9,429 ( 17.1 )	10,593 ( 10.3 )	9,085 ( 14.2 )	7,892 ( 10.1 )	3,999 ( 33.0 )
Cabbage or head lettuce	2,256 ( 36.5 )	8,058 ( 37.6 )	6,363 ( 31.5 )	11,556 ( 31.4 )	8,775 ( 20.4 )	8,645 ( 29.3 )	4,016 ( 32.9 )
Chinese cabbage	1,431 ( 9.5 )	7,634 ( 22.8 )	5,122 ( 22.4 )	11,337 ( 30.0 )	8,040 ( 13.1 )	7,609 ( 8.6 )	3,887 ( 7.5 )
Sansai (Edible wild plants)	2,221 ( 1.2 )	7,699 ( 7.8 )	8,188 ( 2.1 )	10,261 ( 2.0 )	9,043 ( 2.4 )	7,455 ( 1.8 )	3,928 ( 1.4 )
Fungi (enokidake, shiitake, mushroom)	1,476 ( 4.9 )	7,858 ( 11.6 )	5,147 ( 8.1 )	11,179 ( 12.2 )	8,703 ( 6.4 )	8,348 ( 9.4 )	3,978 ( 6.5 )
Potatoes	2,248 ( 14.5 )	8,027 ( 22.4 )	9,713 ( 17.6 )	11,485 ( 40.9 )	9,640 ( 11.6 )	8,534 ( 10.8 )	9,424 ( 13.9 )
Seaweed (algae)	2,251 ( 22.7 )	8,129 ( 37.0 )	9,783 ( 38.3 )	11,525 ( 42.2 )	9,669 ( 30.6 )	8,584 ( 33.8 )	9,385 ( 32.8 )
Pickles	2,247 ( 58.1 )	8,054 ( 63.1 )	9,775 ( 70.3 )	11,429 ( 69.9 )	9,677 ( 62.6 )	8,508 ( 53.0 )	9,354 ( 63.8 )
Tukudani (Preserved foods concocted with say souce)	2,233 ( 2.6 )	7,456 ( 7.8 )	9,559 ( 8.4 )	10,129 ( 6.1 )	9,178 ( 12.6 )	7,678 ( 5.8 )	3,923 ( 5.4 )
Boiled beans	2,240 ( 2.0 )	7,551 ( 6.6 )	8,485 ( 6.8 )	11,127 ( 10.0 )	8,537 ( 5.6 )	8,223 ( 4.6 )	3,967 ( 3.4 )
Tofu (soybean curd)	2,249 ( 31.5 )	8,280 ( 53.1 )	6,383 ( 27.9 )	11,903 ( 32.2 )	9,208 ( 24.8 )	8,872 ( 27.7 )	9,399 ( 30.2 )
Citrus fruits	2,236 ( 16.1 )	7,864 ( 24.5 )	6,335 ( 53.0 )	11,558 ( 58.2 )	8,815 ( 45.7 )	8,492 ( 46.3 )	3,955 ( 23.3 )
Fresh fruit juice (in summer season)	2,222 ( 9.1 )	7,438 ( 19.7 )	4,954 ( 18.3 )	10,246 ( 20.6 )	8,353 ( 21.0 )	7,615 ( 21.4 )	3,895 ( 8.3 )
Other fruits (excluded citrus fruits)	2,222 ( 40.4 )	7,447 ( 37.1 )	6,119 ( 41.7 )	10,723 ( 60.0 )	8,997 ( 31.1 )	7,692 ( 46.7 )	3,888 ( 35.8 )
Sweets	2,247 ( 15.0 )	8,029 ( 22.6 )	6,336 ( 25.8 )	11,521 ( 16.9 )	9,608 ( 15.9 )	8,606 ( 19.0 )	7,292 ( 25.1 )
Coffee	2,254 ( 13.9 )	8,333 ( 17.8 )	9,937 ( 13.2 )	12,179 ( 89.5 )	9,905 ( 41.5 )	9,316 ( 62.2 )	8,902 ( 9.7 )
Black tea	1,580 ( 0.3 )	8,019 ( 0.7 )	8,509 ( 1.2 )	11,095 ( 2.4 )	9,469 ( 1.9 )	8,639 ( 4.3 )	4,007 ( 0.9 )
Green tea	2,238 ( 18.5 )	8,417 ( 52.9 )	8,764 ( 72.9 )	11,994 ( 73.9 )	9,794 ( 76.7 )	8,876 ( 51.5 )	8,100 ( 59.4 )
Oolong tea	1,470 ( 4.3 )	7,138 ( 5.5 )	8,274 ( 4.0 )	10,777 ( 12.6 )	7,997 ( 4.5 )	8,097 ( 12.4 )	3,883 ( 13.4 )

3/day and more, %							
Bowles of rice (at present)	2,244 ( 75.2 )	8,640 ( 78.0 )	9,968 ( 70.3 )	12,022 ( 65.0 )	9,862 ( 77.1 )	9,265 ( 49.1 )	9,329 ( 69.0 )
Bowles of rice (30 years old)	2,210 ( 87.0 )	8,138 ( 89.5 )	9,367 ( 81.9 )	10,873 ( 79.6 )	8,922 ( 87.2 )	8,478 ( 76.7 )	3,855 ( 88.0 )
Bowles of miso soup (at present)	1,903 ( 53.7 )	8,005 ( 48.1 )	7,156 ( 17.6 )	9,095 ( 23.3 )	4,303 ( 7.2 )	4,245 ( 10.4 )	2,535 ( 6.8 )
Bowles of miso soup (30 years old)	1,961 ( 60.7 )	7,688 ( 70.7 )	7,323 ( 36.8 )	8,917 ( 48.8 )	4,761 ( 14.7 )	5,093 ( 17.5 )	2,557 ( 16.4 )

love or like, %							
Taste for salty food	2,255 ( 21.0 )	5,806 ( 30.4 )	9,692 ( 28.3 )	11,548 ( 27.6 )	9,786 ( 25.9 )	8,924 ( 28.3 )	3,966 ( 13.1 )
Taste for fatty food	2,250 ( 14.2 )	5,883 ( 13.2 )	9,750 ( 16.5 )	11,730 ( 17.7 )	9,865 ( 13.7 )	9,192 ( 14.4 )	4,002 ( 5.8 )

Table 4 shows the frequency of food intake according to sex by geographical feature. For both men and women, the percentage of individuals who consumed high amounts of milk and dairy products was lower in plain areas than in seaside and mountainous areas. The percentage of individuals who consumed high amounts of fish was lower in mountainous areas. The percentage of individuals who consumed vegetables was generally lower in seaside areas. The percentage of individuals who consumed high amounts of coffee was higher in seaside areas while the percentage of those who consumed more green tea was highest in mountains/basin areas. The percentages of individuals who consumed more rice and miso soup were lower in seaside areas.

**Table 4.** Sex-specific age-adjusted percentages of higher frequency of foods by geographical feature.

	Men			Women		

	Seaside	Plains	Mountains/Basin	Seaside	Plains	Seaside
					
	No. %	No. %	No. %	No. %	No. %	No. %
3-4/w and more, %						
Beef	4,369 ( 11.4 )	9,723 ( 11.2 )	18,254 ( 7.5 )	8,887 ( 14.4 )	12,701 ( 11.0 )	23,599 ( 8.1 )
Pork (excluding ham and sausages)	4,011 ( 10.3 )	10,001 ( 16.7 )	20,502 ( 25.3 )	8,082 ( 12.6 )	13,284 ( 18.4 )	26,864 ( 25.5 )
Ham	6,366 ( 20.3 )	9,920 ( 14.1 )	21,720 ( 17.6 )	10,909 ( 17.1 )	13,108 ( 13.7 )	28,359 ( 17.9 )
Chiken	4,084 ( 9.8 )	10,343 ( 19.2 )	22,642 ( 19.3 )	8,316 ( 14.7 )	13,796 ( 23.4 )	29,646 ( 22.8 )
Liver	6,402 ( 4.1 )	10,465 ( 5.8 )	16,650 ( 4.0 )	11,154 ( 4.1 )	13,994 ( 5.2 )	21,721 ( 3.9 )
Eggs	7,047 ( 72.1 )	12,553 ( 71.4 )	24,010 ( 71.3 )	12,428 ( 69.0 )	16,476 ( 71.1 )	31,187 ( 71.1 )
Milk	6,768 ( 54.1 )	12,042 ( 51.0 )	23,099 ( 57.1 )	12,083 ( 62.9 )	15,944 ( 56.5 )	30,330 ( 62.4 )
Yogurt	3,086 ( 9.3 )	9,184 ( 5.6 )	19,659 ( 8.8 )	6,820 ( 14.4 )	12,267 ( 8.9 )	25,767 ( 14.2 )
Cheese	3,779 ( 6.8 )	8,682 ( 4.3 )	20,590 ( 7.7 )	7,744 ( 7.4 )	11,574 ( 4.5 )	26,784 ( 8.5 )
Butter	3,732 ( 12.1 )	8,673 ( 8.0 )	20,370 ( 8.7 )	7,567 ( 13.6 )	11,576 ( 9.8 )	26,487 ( 10.6 )
Margarine	6,225 ( 24.9 )	9,497 ( 13.7 )	17,792 ( 13.5 )	10,942 ( 37.6 )	12,524 ( 19.4 )	23,687 ( 19.1 )
Deep-fried foods or tempura	4,191 ( 15.2 )	9,020 ( 25.3 )	19,518 ( 23.8 )	8,620 ( 14.0 )	12,144 ( 24.7 )	25,971 ( 23.0 )
Fried vegetables	4,278 ( 30.3 )	8,887 ( 38.4 )	20,111 ( 34.0 )	8,912 ( 32.9 )	12,027 ( 42.0 )	26,746 ( 39.0 )
Raw fish	7,029 ( 62.6 )	12,119 ( 60.8 )	21,181 ( 56.1 )	12,306 ( 62.2 )	15,845 ( 63.1 )	27,927 ( 58.2 )
*Kamaboko* (fish paste)	3,396 ( 12.6 )	8,532 ( 17.5 )	20,306 ( 11.8 )	7,270 ( 13.5 )	11,222 ( 19.0 )	26,332 ( 14.3 )
*Himono* (Dried fish or salted fish)	4,069 ( 17.0 )	10,163 ( 37.1 )	20,166 ( 26.4 )	8,131 ( 16.2 )	13,507 ( 37.6 )	26,713 ( 27.4 )

5/w and more, %						
Spinach or garland chrysanthemumm	4,360 ( 25.8 )	9,734 ( 31.5 )	22,999 ( 29.7 )	8,926 ( 35.3 )	12,937 ( 37.3 )	29,820 ( 34.0 )
Carrot or pumpkin	4,164 ( 7.9 )	9,669 ( 11.3 )	20,709 ( 16.4 )	8,720 ( 17.4 )	13,018 ( 18.0 )	27,963 ( 25.9 )
Tomatoes	4,071 ( 8.3 )	10,309 ( 13.4 )	22,371 ( 10.6 )	8,341 ( 14.9 )	13,628 ( 18.7 )	29,173 ( 16.7 )
Cabbage or head lettuce	4,345 ( 19.6 )	9,544 ( 20.7 )	20,766 ( 24.2 )	9,025 ( 28.9 )	12,904 ( 29.3 )	27,740 ( 31.5 )
Chinese cabbage	3,996 ( 8.1 )	8,420 ( 12.3 )	19,810 ( 19.9 )	8,139 ( 8.6 )	10,975 ( 15.0 )	25,946 ( 23.4 )
*Sansai* (Edible wild plants)	3,911 ( 0.6 )	10,149 ( 4.5 )	21,283 ( 1.9 )	7,900 ( 0.8 )	13,385 ( 5.4 )	27,510 ( 2.2 )
Fungi (enokidake, shiitake, mushroom)	4,239 ( 4.4 )	8,881 ( 6.3 )	19,556 ( 7.1 )	8,781 ( 8.6 )	11,947 ( 8.4 )	25,961 ( 10.0 )
Potatoes	6,886 ( 8.2 )	12,173 ( 7.5 )	23,310 ( 20.7 )	12,290 ( 13.8 )	16,137 ( 12.2 )	30,644 ( 26.8 )
Seaweed (algae)	6,953 ( 21.8 )	12,362 ( 22.3 )	23,499 ( 30.2 )	12,288 ( 32.2 )	16,246 ( 30.2 )	30,792 ( 39.6 )
Pickles	6,852 ( 52.2 )	12,345 ( 59.7 )	23,465 ( 62.2 )	12,146 ( 55.6 )	16,255 ( 64.0 )	30,643 ( 67.3 )
*Tukudani* (Preserved foods concocted with say souce)	4,044 ( 4.3 )	10,078 ( 8.5 )	22,044 ( 7.0 )	8,217 ( 5.2 )	13,353 ( 10.0 )	28,586 ( 7.4 )
Boiled beans	4,137 ( 2.8 )	9,557 ( 4.4 )	21,772 ( 5.5 )	8,624 ( 3.9 )	12,884 ( 5.9 )	28,622 ( 7.4 )
Tofu (soybean curd)	7,057 ( 24.0 )	11,798 ( 27.1 )	21,306 ( 27.9 )	12,488 ( 27.6 )	15,509 ( 32.0 )	28,297 ( 34.9 )
Citrus fruits	4,266 ( 21.3 )	9,357 ( 19.7 )	20,603 ( 31.6 )	8,903 ( 44.3 )	12,746 ( 33.7 )	27,606 ( 47.1 )
Fresh fruit juice (in summer season)	4,018 ( 12.4 )	9,123 ( 15.7 )	18,668 ( 15.0 )	8,083 ( 18.4 )	12,224 ( 20.1 )	24,416 ( 18.2 )
Other fruits (excluded citrus fruits)	4,014 ( 23.7 )	9,623 ( 18.0 )	19,671 ( 32.6 )	8,131 ( 45.5 )	12,778 ( 31.7 )	26,179 ( 48.4 )
Sweets	6,864 ( 14.0 )	10,215 ( 13.3 )	20,663 ( 13.9 )	12,223 ( 20.4 )	13,745 ( 19.8 )	27,671 ( 20.0 )
Coffee	7,142 ( 38.2)	12,373 ( 29.9 )	24,265 ( 36.6 )	12,801 ( 42.1 )	16,341 ( 31.1 )	31,684 ( 30.1 )
Black tea	4,321 ( 2.6 )	10,210 ( 1.1 )	22,096 ( 1.5 )	8,958 ( 3.9 )	13,499 ( 1.2)	28,861 ( 1.8 )
Green tea	7,043 ( 52.2 )	11,775 ( 58.4)	23,491 ( 71.0 )	12,515 ( 53.6 )	15,272 ( 56.9 )	30,396 ( 72.1 )
Oolong tea	4,160 ( 7.0)	8,531 ( 3.7 )	21,545 ( 5.3 )	8,476 ( 12.0 )	11,125 ( 5.6 )	28,035 ( 8.6 )

3/day and more, %						
Bowles of rice (at present)	7,145 ( 69.5 )	12,713 ( 77.3 )	24,460 ( 76.0 )	12,743 ( 57.2 )	16,734 ( 74.8 )	31,853 ( 69.2 )
Bowles of rice (30 years old)	4,312 ( 84.0 )	10,266 ( 90.7 )	22,910 ( 86.9 )	8,806 ( 78.1 )	13,521 ( 87.6 )	29,516 ( 83.0 )
Bowles of miso soup (at present)	2,355 ( 18.3 )	7,909 ( 42.6 )	18,570 ( 36.2 )	4,054 ( 8.6 )	10,202 ( 34.5 )	22,986 ( 23.1 )
Bowles of miso soup (30 years old)	2,724 ( 25.3 )	7,913 ( 51.8 )	18,324 ( 53.0 )	5,058 ( 16.4 )	10,308 ( 45.5 )	22,934 ( 44.3 )

love or like, %						
Taste for salty food	4,491 ( 40.9 )	10,977 ( 41.4 )	22,159 ( 42.5 )	9,182 ( 25.6 )	14,436 ( 27.2 )	28,359 ( 26.3 )
Taste for fatty food	4,548 ( 28.1 )	11,021 ( 26.7 )	22,275 ( 31.9 )	9,447 ( 13.2 )	14,548 ( 14.2 )	28,677 ( 15.3 )

Table 5 shows the frequency of estimated major nutrient intake according to sex groups by geographical area. For both men and women, the intake values of mean total energy and other major nutrient except animal fat were higher in Tohoku, and generally lower in Chugoku.

**Table 4.** Sex-specific age-adjusted means and standard deviations of nutrient intake by geographical area.

		Hokkaido	Tohoku	Kanto	Chubu	Kinki	Chugoku	Kyusyu
Men								
No of subjects		786	3,972	3,611	6,758	5,092	3,161	1,006
Total weight	g/day	1435 ( 18 )	1788 ( 8 )	1739 ( 8 )	1858 ( 6 )	1710 ( 7 )	1481 ( 9 )	1710 ( 16 )
Total enargy	kcal/day	1587 ( 16 )	1872 ( 7 )	1570 ( 8 )	1634 ( 6 )	1629 ( 6 )	1464 ( 8 )	1593 ( 14 )
Water	g/day	1120 ( 15 )	1418 ( 7 )	1427 ( 7 )	1533 ( 5 )	1383 ( 6 )	1200 ( 8 )	1391 ( 14 )
Animal protein	g/day	23.8 ( 0.3 )	28.5 ( 0.2 )	24.6 ( 0.2 )	26.8 ( 0.1 )	24.1 ( 0.1 )	23.2 ( 0.2 )	25.2 ( 0.3 )
Vegetable protein	g/day	29.3 ( 0.3 )	35.3 ( 0.1 )	28.1 ( 0.1 )	28.8 ( 0.1 )	26.1 ( 0.1 )	23.6 ( 0.2 )	26.7 ( 0.3 )
Total protein	g/day	53.2 ( 0.5 )	63.9 ( 0.2 )	52.7 ( 0.3 )	55.6 ( 0.2 )	50.2 ( 0.2 )	46.8 ( 0.3 )	51.9 ( 0.5 )
Animal fat	g/day	12.1 ( 0.2 )	13.2 ( 0.1 )	13.0 ( 0.1 )	14.3 ( 0.1 )	12.4 ( 0.1 )	13.0 ( 0.1 )	12.5 ( 0.2 )
Vegetable fat	g/day	15.1 ( 0.2 )	18.0 ( 0.1 )	14.3 ( 0.1 )	14.5 ( 0.1 )	11.8 ( 0.1 )	12.2 ( 0.1 )	12.8 ( 0.2 )
Total fat	g/day	30.7 ( 0.4 )	35.6 ( 0.2 )	30.6 ( 0.2 )	32.5 ( 0.1 )	27.8 ( 0.1 )	28.3 ( 0.2 )	28.9 ( 0.3 )
Carbohydrate	g/day	215.8 ( 2.6 )	251.1 ( 1.2 )	212.0 ( 1.2 )	220.4 ( 0.9 )	235.6 ( 1.0 )	193.5 ( 1.3 )	224.1 ( 2.3 )
Crude fiber	g/day	3.07 (0.04 )	3.76 (0.02 )	3.04 (0.02 )	3.17 (0.01 )	2.42 (0.01 )	2.37 (0.02 )	2.61 (0.03 )
Ash	g/day	12.2 ( 0.1 )	14.9 ( 0.1 )	12.3 ( 0.1 )	12.8 ( 0.0 )	9.7 ( 0.1 )	9.9 ( 0.1 )	10.3 ( 0.1 )
Calcium	mg/day	450.7 ( 5.5 )	518.5 ( 2.4 )	457.1 ( 2.6 )	493.6 ( 1.9 )	386.1 ( 2.2 )	421.8 ( 2.8 )	390.9 ( 4.8 )
Phosphate	mg/day	744.9 ( 7.8 )	879.7 ( 3.5 )	748.4 ( 3.6 )	802.0 ( 2.7 )	700.0 ( 3.0 )	678.9 ( 3.9 )	707.7 ( 6.9 )
Iron	mg/day	7.93 (0.09 )	10.04 (0.04 )	8.26 (0.04 )	8.38 (0.03 )	6.69 (0.04 )	6.62 (0.05 )	7.32 (0.08 )
Sodium	mg/day	2427 ( 31 )	2989 ( 14 )	2339 ( 14 )	2316 ( 11 )	1623 ( 12 )	1727 ( 16 )	1774 ( 27 )
Potassium	mg/day	1863 ( 21 )	2214 ( 9 )	1939 ( 10 )	2127 ( 7 )	1701 ( 8 )	1692 ( 11 )	1741 ( 18 )
Retinol	*μ*g/day	427 ( 30 )	762 ( 13 )	573 ( 14 )	560 ( 10 )	545 ( 12 )	531 ( 15 )	693 ( 26 )
Carotin	*μ*g/day	1929 ( 31 )	2320 ( 14 )	2001 ( 14 )	1970 ( 10 )	1551 ( 12 )	1611 ( 15 )	1783 ( 27 )
Vitamin A	IU/day	2509 ( 104 )	3838 ( 46 )	3034 ( 48 )	2977 ( 35 )	2691 ( 41 )	2681 ( 52 )	3313 ( 92 )
Vitamin B_1_	mg/day	0.76 (0.01 )	0.91 (0.00 )	0.78 (0.00 )	0.83 (0.00 )	0.69 (0.00 )	0.65 (0.00 )	0.72 (0.01 )
Vitamin B_2_	mg/day	0.92 (0.01 )	1.14 (0.01 )	1.03 (0.01 )	1.10 (0.00 )	0.94 (0.00 )	0.94 (0.01 )	0.99 (0.01 )
Niacine	mg/day	10.5 ( 0.1 )	13.0 ( 0.1 )	10.7 ( 0.1 )	11.5 ( 0.0 )	10.1 ( 0.0 )	9.2 ( 0.1 )	10.7 ( 0.1 )
Vitamin C	mg/day	91.3 ( 1.4 )	109.6 ( 0.6 )	102.6 ( 0.7 )	109.3 ( 0.5 )	87.0 ( 0.5 )	84.5 ( 0.7 )	87.8 ( 1.2 )
Salt	g/day	6.04 (0.08 )	7.45 (0.03 )	5.81 (0.04 )	5.74 (0.03 )	4.00 (0.03 )	4.26 (0.04 )	4.38 (0.07 )
Cholesterol	mg/day	219.4 ( 3.3 )	270.2 ( 1.5 )	239.3 ( 1.5 )	254.3 ( 1.1 )	219.3 ( 1.3 )	219.9 ( 1.7 )	226.7 ( 2.9 )
*α*-tocopherol	mg/day	3.94 (0.04 )	4.67 (0.02 )	3.91 (0.02 )	4.07 (0.01 )	3.60 (0.02 )	3.38 (0.02 )	3.83 (0.04 )
*β*-tocopherol	mg/day	0.15 (0.00 )	0.18 (0.00 )	0.13 (0.00 )	0.13 (0.00 )	0.08 (0.00 )	0.10 (0.00 )	0.10 (0.00 )
*γ*-tocopherol	mg/day	4.85 (0.07 )	6.18 (0.03 )	4.48 (0.03 )	4.45 (0.02 )	2.71 (0.03 )	3.23 (0.03 )	3.43 (0.06 )
*δ*-tocopherol	mg/day	1.99 (0.03 )	2.57 (0.01 )	1.82 (0.01 )	1.79 (0.01 )	1.03 (0.01 )	1.25 (0.01 )	1.34 (0.03 )
Vitamin E	mg/day	4.23 (0.04 )	5.03 (0.02 )	4.17 (0.02 )	4.32 (0.01 )	3.75 (0.02 )	3.56 (0.02 )	4.02 (0.04 )
Total fatty acids	mg/day	26.4 ( 0.3 )	30.8 ( 0.1 )	26.5 ( 0.1 )	28.0 ( 0.1 )	23.9 ( 0.1 )	24.2 ( 0.2 )	25.0 ( 0.3 )
Saturted fatty acids	mg/day	8.71 (0.12 )	9.59 (0.05 )	8.84 (0.05 )	9.67 (0.04 )	8.58 (0.05 )	8.88 (0.06 )	8.43 (0.10 )
Monounsaturted fatty acids	mg/day	9.16 (0.12 )	10.71 (0.05 )	9.36 (0.06 )	9.85 (0.04 )	8.56 (0.05 )	8.50 (0.06 )	9.06 (0.11 )
Polyunsaturted fatty acids	mg/day	8.47 (0.10 )	10.43 (0.04 )	8.24 (0.05 )	8.34 (0.03 )	6.67 (0.04 )	6.64 (0.05 )	7.46 (0.09 )
n3-fatty acids	mg/day	1.70 (0.02 )	2.08 (0.01 )	1.62 (0.01 )	1.71 (0.01 )	1.42 (0.01 )	1.37 (0.01 )	1.55 (0.02 )
n6-fatty acids	mg/day	6.77 (0.08 )	8.34 (0.03 )	6.62 (0.04 )	6.64 (0.03 )	5.26 (0.03 )	5.27 (0.04 )	5.91 (0.07 )

Women								
No of subjects		1,358	6,032	4,631	8,740	6,920	6,048	3,764
Total weight	g/day	1363 ( 12 )	1653 ( 6 )	1690 ( 6 )	1802 ( 5 )	1658 ( 5 )	1497 ( 6 )	1647 ( 7 )
Total enargy	kcal/day	1271 ( 9 )	1438 ( 4 )	1294 ( 5 )	1320 ( 4 )	1366 ( 4 )	1224 ( 4 )	1289 ( 5 )
Water	g/day	1078 ( 11 )	1329 ( 5 )	1398 ( 6 )	1504 ( 4 )	1349 ( 5 )	1224 ( 5 )	1357 ( 6 )
Animal protein	g/day	24.4 ( 0.3 )	28.5 ( 0.1 )	25.5 ( 0.1 )	27.1 ( 0.1 )	24.6 ( 0.1 )	23.6 ( 0.1 )	27.0 ( 0.2 )
Vegetable protein	g/day	26.3 ( 0.2 )	31.0 ( 0.1 )	26.2 ( 0.1 )	26.4 ( 0.1 )	24.9 ( 0.1 )	22.5 ( 0.1 )	24.3 ( 0.1 )
Total protein	g/day	50.7 ( 0.4 )	59.5 ( 0.2 )	51.7 ( 0.2 )	53.4 ( 0.2 )	49.5 ( 0.2 )	46.1 ( 0.2 )	51.4 ( 0.2 )
Animal fat	g/day	12.3 ( 0.2 )	13.2 ( 0.1 )	13.8 ( 0.1 )	14.6 ( 0.1 )	12.9 ( 0.1 )	13.7 ( 0.1 )	13.8 ( 0.1 )
Vegetable fat	g/day	14.5 ( 0.1 )	16.8 ( 0.1 )	14.2 ( 0.1 )	14.2 ( 0.1 )	12.2 ( 0.1 )	12.6 ( 0.1 )	12.9 ( 0.1 )
Total fat	g/day	30.6 ( 0.3 )	34.5 ( 0.1 )	31.3 ( 0.2 )	32.5 ( 0.1 )	28.6 ( 0.1 )	29.2 ( 0.1 )	30.5 ( 0.2 )
Carbohydrate	g/day	188.7 ( 1.5 )	212.2 ( 0.7 )	193.8 ( 0.8 )	195.4 ( 0.6 )	217.7 ( 0.7 )	184.1 ( 0.7 )	193.6 ( 0.9 )
Crude fiber	g/day	3.03 (0.02 )	3.64 (0.01 )	3.13 (0.01 )	3.19 (0.01 )	2.53 (0.01 )	2.47 (0.01 )	2.71 (0.01 )
Ash	g/day	11.8 ( 0.1 )	14.2 ( 0.0 )	12.4 ( 0.1 )	12.7 ( 0.0 )	10.0 ( 0.0 )	9.9 ( 0.0 )	10.6 ( 0.1 )
Calcium	mg/day	455.0 ( 4.1 )	514.3 ( 1.9 )	476.8 ( 2.2 )	504.2 ( 1.6 )	407.1 ( 1.8 )	439.2 ( 2.0 )	432.0 ( 2.5 )
Phosphate	mg/day	721.5 ( 5.6 )	832.9 ( 2.7 )	748.7 ( 3.0 )	785.3 ( 2.2 )	701.3 ( 2.5 )	679.1 ( 2.7 )	719.7 ( 3.4 )
Iron	mg/day	7.62 (0.07 )	9.50 (0.03 )	8.23 (0.04 )	8.22 (0.03 )	6.87 (0.03 )	6.66 (0.03 )	7.56 (0.04 )
Sodium	mg/day	2219 ( 21 )	2745 ( 10 )	2241 ( 11 )	2203 ( 8 )	1625 ( 9 )	1590 ( 10 )	1733 ( 13 )
Potassium	mg/day	1922 ( 15 )	2230 ( 7 )	2056 ( 8 )	2195 ( 6 )	1811 ( 7 )	1823 ( 7 )	1903 ( 9 )
Retinol	*μ*g/day	360 ( 23 )	551 ( 11 )	533 ( 13 )	490 ( 9 )	536 ( 10 )	541 ( 11 )	737 ( 14 )
Carotin	*μ*g/day	2168 ( 23 )	2480 ( 11 )	2246 ( 13 )	2130 ( 9 )	1777 ( 10 )	1875 ( 11 )	2120 ( 14 )
Vitamin A	IU/day	2418 ( 80 )	3222 ( 38 )	3036 ( 43 )	2831 ( 32 )	2788 ( 36 )	2863 ( 38 )	3648 ( 48 )
Vitamin B_1_	mg/day	0.75 (0.01 )	0.87 (0.00 )	0.80 (0.00 )	0.82 (0.00 )	0.71 (0.00 )	0.68 (0.00 )	0.75 (0.00 )
Vitamin B_2_	mg/day	0.92 (0.01 )	1.09 (0.00 )	1.06 (0.01 )	1.11 (0.00 )	0.97 (0.00 )	0.99 (0.00 )	1.06 (0.01 )
Niacine	mg/day	10.3 ( 0.1 )	12.4 ( 0.0 )	10.8 ( 0.0 )	11.3 ( 0.0 )	10.1 ( 0.0 )	9.2 ( 0.0 )	10.9 ( 0.1 )
Vitamin C	mg/day	102.2 ( 1.0 )	118.4 ( 0.5 )	117.0 ( 0.6 )	120.5 ( 0.4 )	99.8 ( 0.5 )	101.8 ( 0.5 )	101.7 ( 0.6 )
Salt	g/day	5.51 (0.05 )	6.83 (0.03 )	5.56 (0.03 )	5.46 (0.02 )	4.00 (0.02 )	3.91 (0.03 )	4.28 (0.03 )
Cholesterol	mg/day	221.1 ( 2.5 )	263.6 ( 1.2 )	243.0 ( 1.4 )	252.4 ( 1.0 )	221.5 ( 1.1 )	217.7 ( 1.2 )	239.3 ( 1.5 )
*α*-tocopherol	mg/day	3.96 ( 0.03 )	4.51 (0.01 )	3.97 (0.02 )	3.99 (0.01 )	3.65 (0.01 )	3.48 (0.01 )	3.92 (0.02 )
*β*-tocopherol	mg/day	0.14 ( 0.00 )	0.16 (0.00 )	0.13 (0.00 )	0.13 (0.00 )	0.09 (0.00 )	0.10 (0.00 )	0.10 (0.00 )
*γ*-tocopherol	mg/day	4.41 ( 0.04 )	5.62 (0.02 )	4.22 (0.02 )	4.17 (0.02 )	2.71 (0.02 )	2.93 (0.02 )	3.39 (0.03 )
*δ*-tocopherol	mg/day	1.74 (0.02 )	2.30 (0.01 )	1.67 (0.01 )	1.64 (0.01 )	1.00 (0.01 )	1.06 (0.01 )	1.28 (0.01 )
Vitamin E	mg/day	4.21 (0.03 )	4.84 (0.01 )	4.21 (0.02 )	4.23 (0.01 )	3.79 (0.01 )	3.64 (0.01 )	4.11 (0.02 )
Total fatty acids	mg/day	26.0 ( 0.2 )	29.7 ( 0.1 )	26.8 ( 0.1 )	27.8 ( 0.1 )	24.3 ( 0.1 )	24.4 ( 0.1 )	25.9 ( 0.1 )
Saturted fatty acids	mg/day	8.68 (0.09 )	9.33 (0.04 )	9.08 (0.05 )	9.66 (0.03 )	8.72 (0.04 )	9.12 (0.04 )	8.95 (0.05 )
Monounsaturted fatty acids	mg/day	9.18 (0.09 )	10.52 (0.04 )	9.60 (0.05 )	9.89 (0.04 )	8.76 (0.04 )	8.69 (0.04 )	9.53 (0.05 )
Polyunsaturted fatty acids	mg/day	8.07 (0.07 )	9.82 (0.03 )	8.07 (0.04 )	8.09 (0.03 )	6.68 (0.03 )	6.42 (0.03 )	7.39 (0.04 )
n3-fatty acids	mg/day	1.71 (0.02 )	2.06 (0.01 )	1.63 (0.01 )	1.71 (0.01 )	1.45 (0.01 )	1.35 (0.01 )	1.60 (0.01 )
n6-fatty acids	mg/day	6.35 (0.05 )	7.75 (0.03 )	6.43 (0.03 )	6.38 (0.02 )	5.23 (0.02 )	5.08 (0.03 )	5.79 (0.03 )

Standard deviations in parentheses.

Table 6 shows the frequency of estimated major nutrient intake according to sex groups by geographical feature. For both men and women, the mean intake values of total energy and other major nutrient except for animal fat were lowest in seaside areas while the mean animal fat intake was highest in mountainous areas. The mean intake levels of retinol, carotene and vitamin A were highest in plain areas, and mean vitamin C intake was higher in mountainous areas.

**Table 6.** Sex-specific age-adjusted means and standard deviations of nutrient intake by geographical feature.

		Men			Women		
	
		Seaside	Plains	Mountains/Basin	Seaside	Plains	Mountains/Basin
No of subjects		3,124	6,723	14,539	6,633	9,634	21,226
Total weight	g/day	1495 ( 9 )	1745 ( 6 )	1773 ( 4 )	1496 ( 6 )	1651 ( 5 )	1710 ( 3 )
Total enargy	kcal/day	1467 ( 8 )	1744 ( 6 )	1624 ( 4 )	1223 ( 4 )	1401 ( 3 )	1320 ( 2 )
Water	g/day	1210 ( 8 )	1398 ( 5 )	1450 ( 4 )	1223 ( 5 )	1335 ( 4 )	1412 ( 3 )
Animal protein	g/day	22.8 ( 0.2 )	26.1 ( 0.1 )	25.9 ( 0.1 )	23.7 ( 0.1 )	26.7 ( 0.1 )	26.4 ( 0.1 )
Vegetable protein	g/day	23.9 ( 0.2 )	30.3 ( 0.1 )	28.6 ( 0.1 )	22.7 ( 0.1 )	27.6 ( 0.1 )	26.3 ( 0.1 )
Total protein	g/day	46.7 ( 0.3 )	56.4 ( 0.2 )	54.5 ( 0.1 )	46.4 ( 0.2 )	54.2 ( 0.1 )	52.7 ( 0.1 )
Animal fat	g/day	12.6 ( 0.1 )	12.7 ( 0.1 )	13.6 ( 0.0 )	13.4 ( 0.1 )	13.1 ( 0.1 )	14.0 ( 0.0 )
Vegetable fat	g/day	12.2 ( 0.1 )	14.6 ( 0.1 )	14.3 ( 0.0 )	12.6 ( 0.1 )	14.3 ( 0.1 )	14.1 ( 0.0 )
Total fat	g/day	27.9 ( 0.2 )	31.3 ( 0.1 )	31.5 ( 0.1 )	29.1 ( 0.1 )	31.4 ( 0.1 )	31.7 ( 0.1 )
Carbohydrate	g/day	197.7 ( 1.3 )	243.4 ( 0.9 )	220.4 ( 0.6 )	184.1 ( 0.7 )	214.8 ( 0.6 )	197.8 ( 0.4 )
Crude fiber	g/day	2.35 ( 0.02 )	3.02 ( 0.01 )	3.06 ( 0.01 )	2.48 ( 0.01 )	3.03 ( 0.01 )	3.08 ( 0.01 )
Ash	g/day	9.66 ( 0.08 )	12.11 ( 0.05 )	12.32 ( 0.03 )	9.84 ( 0.05 )	12.00 ( 0.04 )	12.13 ( 0.03 )
Calcium	mg/day	404.8 ( 2.9 )	451.8 ( 2.0 )	467.1 ( 1.3 )	428.7 ( 1.9 )	463.4 ( 1.6 )	477.1 ( 1.1 )
Phosphate	mg/day	667.2 ( 4.0 )	785.8 ( 2.7 )	774.2 ( 1.9 )	673.8 ( 2.6 )	767.7 ( 2.1 )	760.3 ( 1.5 )
Iron	mg/day	6.50 ( 0.05 )	8.21 ( 0.03 )	8.21 ( 0.02 )	6.68 ( 0.03 )	8.09 ( 0.03 )	8.08 ( 0.02 )
Sodium	mg/day	1664 ( 17 )	2270 ( 12 )	2265 ( 8 )	1583 ( 11 )	2165 ( 9 )	2130 ( 6 )
Potassium	mg/day	1671 ( 11 )	1928 ( 7 )	2010 ( 5 )	1815 ( 7 )	2005 ( 6 )	2079 ( 4 )
Retinol	*μ*g/day	488 ( 15 )	661 ( 10 )	578 ( 7 )	528 ( 11 )	583 ( 9 )	527 ( 6 )
Carotin	*μ*g/day	1605 ( 16 )	1883 ( 11 )	1952 ( 7 )	1919 ( 11 )	2095 ( 9 )	2150 ( 6 )
Vitamin A	IU/day	2533 ( 53 )	3260 ( 36 )	3024 ( 24 )	2842 ( 37 )	3120 ( 30 )	2966 ( 20 )
Vitamin B_1_	mg/day	0.65 ( 0.00 )	0.78 ( 0.00 )	0.80 ( 0.00 )	0.69 ( 0.00 )	0.78 ( 0.00 )	0.80 ( 0.00 )
Vitamin B_2_	mg/day	0.91 ( 0.01 )	1.04 ( 0.00 )	1.05 ( 0.00 )	0.97 ( 0.00 )	1.04 ( 0.00 )	1.07 ( 0.00 )
Niacine	mg/day	9.21 ( 0.07 )	11.30 ( 0.04 )	11.17 ( 0.03 )	9.43 ( 0.04 )	11.14 ( 0.03 )	11.04 ( 0.02 )
Vitamin C	mg/day	84.7 ( 0.7 )	95.9 ( 0.5 )	103.5 ( 0.3 )	101.7 ( 0.5 )	107.7 ( 0.4 )	114.2 ( 0.3 )
Salt	g/day	4.11 ( 0.04 )	5.63 ( 0.03 )	5.62 ( 0.02 )	3.90 ( 0.03 )	5.37 ( 0.02 )	5.28 ( 0.01 )
Cholesterol	mg/day	211.0 ( 1.7 )	242.9 ( 1.2 )	246.0 ( 0.8 )	213.6 ( 1.2 )	243.0 ( 1.0 )	245.6 ( 0.6 )
*α*-tocopherol	mg/day	3.42 ( 0.02 )	4.06 ( 0.01 )	3.99 ( 0.01 )	3.56 ( 0.01 )	4.03 ( 0.01 )	3.98 ( 0.01 )
*β*-tocopherol	mg/day	0.10 ( 0.00 )	0.13 ( 0.00 )	0.13 ( 0.00 )	0.10 ( 0.00 )	0.12 ( 0.00 )	0.12 ( 0.00 )
*γ*-tocopherol	mg/day	3.14 ( 0.04 )	4.27 ( 0.03 )	4.37 ( 0.02 )	2.97 ( 0.02 )	4.03 ( 0.02 )	4.08 ( 0.01 )
*δ*-tocopherol	mg/day	1.20 ( 0.02 )	1.73 ( 0.01 )	1.76 ( 0.01 )	1.08 ( 0.01 )	1.59 ( 0.01 )	1.60 ( 0.01 )
Vitamin E	mg/day	3.59 ( 0.02 )	4.31 ( 0.02 )	4.25 ( 0.01 )	3.71 ( 0.01 )	4.26 ( 0.01 )	4.21 ( 0.01 )
Total fatty acids	mg/day	23.8 ( 0.2 )	27.1 ( 0.1 )	27.2 ( 0.1 )	24.2 ( 0.1 )	26.9 ( 0.1 )	27.1 ( 0.1 )
Saturted fatty acids	mg/day	8.64 ( 0.06 )	9.08 ( 0.04 )	9.24 ( 0.03 )	8.91 ( 0.04 )	9.08 ( 0.03 )	9.28 ( 0.02 )
Monounsaturted fatty acids	mg/day	8.41 ( 0.06 )	9.50 ( 0.04 )	9.59 ( 0.03 )	8.70 ( 0.04 )	9.57 ( 0.03 )	9.70 ( 0.02 )
Polyunsaturted fatty acids	mg/day	6.59 ( 0.05 )	8.37 ( 0.04 )	8.24 ( 0.02 )	6.50 ( 0.03 )	8.13 ( 0.03 )	8.00 ( 0.02 )
n3-fatty acids	mg/day	1.37 ( 0.01 )	1.72 ( 0.01 )	1.67 ( 0.01 )	1.39 ( 0.01 )	1.74 ( 0.01 )	1.67 ( 0.00 )
n6-fatty acids	mg/day	5.22 ( 0.04 )	6.65 ( 0.03 )	6.57 ( 0.02 )	5.11 ( 0.03 )	6.39 ( 0.02 )	6.32 ( 0.01 )

Standard deviations in parentheses.

## DISCUSSION

We found the consumption of certain foods and nutrients varied according to age and sex in the JACC Study cohort. Compared with men, women were more likely to consume vegetables, seaweed, fruits, sweets, oolong tea, western-style breakfast, but less likely to have rice and miso soup. These sex differences suggest that women has more westernized dietary pattern than men. Women also reported less preference of salty foods and fatty foods than men, indicating that women attempt to have healthier diet than men.^2^ Women had higher mean intakes of carotene and vitamin C, and lower intake of total energy, carbohydrate and sodium than men. These sex difference were compatible with the findings of the National Nutrition Surveys.^2^

The frequency of consumption of beef, chicken, dairy products, fresh fish, fish products, rice, miso soup increased with age in men, and that of vegetables, seaweed, beans, tofu, fruits, sweets, green tea increased with age in both men and women. These age-related changes in food consumption suggest that older men had a mixture of unhealthy and healthy dietary pattern while older women generally had a healthier dietary pattern compared with younger persons. The exception was that women aged 70-79 years had low mean intakes of calcium, retinol, and vitamins A. As for potential problems related to major nutrient intake, men aged 40-49 years had the lowest mean intake values of crude fiber, calcium, iron, retinol, carotene, vitamins A, C and E than men of other age groups. Likewise, women of the same age group also had the lowest mean intake values of crude fiber and iron than women of other age groups. These potential problems had not been highlighted in previous nutrition surveys, and should be confirmed by additional analysis of the National Nutrition Surveys or other studies.

Our analysis showed substantial differences in the frequency of food intake and mean major nutrient intake by geographical area and feature. Similar differences were observed in a previous nutrition study based on 24-hour dietary recall. Residents in seaside areas of Akita and Kochi prefecture had higher intake of fish and shellfish than residents in Osaka prefecture.^3^ These differences were attributable mostly to the accessibility to foods and local culture, some of which may contribute to regional differences in mortality from certain causes. Previous ecological analyses of the data of the National Nutrition Surveys in Japan and mortality statistics indicated that miso, pickled vegetables, soy products and fish (traditional Japanese diets) were positively associated with mortality due to cerebrovascular disease, while intakes of beef, eggs, butter, margarine and wheat (western-style diets) were inversely associated with mortality.^4^

Our findings are potentially useful for the development of hypothesis and interpretation of the relationships between food types and nutrient and the risk of mortality from various diseases.

## MEMBER LIST OF THE JACC STUDY GROUP

The present investigators involved, with the co-authorship of this paper, in the JACC Study and their affiliations are as follows: Dr. Akiko Tamakoshi (present chairman of the study group), Nagoya University Graduate School of Medicine; Dr. Mitsuru Mori, Sapporo Medical University School of Medicine; Dr. Yutaka Motohashi, Akita University School of Medicine; Dr. Ichiro Tsuji, Tohoku University Graduate School of Medicine; Dr. Yosikazu Nakamura, Jichi Medical School; Dr. Hiroyasu Iso, Institute of Community Medicine, University of Tsukuba; Dr. Haruo Mikami, Chiba Cancer Center; Dr. Yutaka Inaba, Juntendo University School of Medicine; Dr. Yoshiharu Hoshiyama, University of Human Arts and Sciences; Dr. Hiroshi Suzuki, Niigata University School of Medicine; Dr. Hiroyuki Shimizu, Gifu University School of Medicine; Dr. Hideaki Toyoshima, Nagoya University Graduate School of Medicine; Dr. Kenji Wakai, Aichi Cancer Center Research Institute; Dr. Shinkan Tokudome, Nagoya City University Graduate School of Medical Sciences; Dr. Yoshinori Ito, Fujita Health University School of Health Sciences; Dr. Shuji Hashimoto, Fujita Health University School of Medicine; Dr. Shogo Kikuchi, Aichi Medical University School of Medicine; Dr. Akio Koizumi, Graduate School of Medicine and Faculty of Medicine, Kyoto University; Dr. Takashi Kawamura, Kyoto University Center for Student Health; Dr. Yoshiyuki Watanabe, Kyoto Prefectural University of Medicine Graduate School of Medical Science; Dr. Tsuneharu Miki, Graduate School of Medical Science, Kyoto Prefectural University of Medicine; Dr. Chigusa Date, Faculty of Human Environmental Sciences, Mukogawa Women’s University ; Dr. Kiyomi Sakata, Wakayama Medical University; Dr. Takayuki Nose, Tottori University Faculty of Medicine; Dr. Norihiko Hayakawa, Research Institute for Radiation Biology and Medicine, Hiroshima University; Dr. Takesumi Yoshimura, Fukuoka Institute of Health and Environmental Sciences; Dr. Akira Shibata, Kurume University School of Medicine; Dr. Naoyuki Okamoto, Kanagawa Cancer Center; Dr. Hideo Shio, Moriyama Municipal Hospital; Dr. Yoshiyuki Ohno, Asahi Rosai Hospital; Dr. Tomoyuki Kitagawa, Cancer Institute of the Japanese Foundation for Cancer Research; Dr. Toshio Kuroki, Gifu University; and Dr. Kazuo Tajima, Aichi Cancer Center Research Institute.
